# Two-step structural changes in M3 muscarinic receptor activation rely on the coupled G_q_ protein cycle

**DOI:** 10.1038/s41467-023-36911-4

**Published:** 2023-03-08

**Authors:** Yong-Seok Kim, Jun-Hee Yeon, Woori Ko, Byung-Chang Suh

**Affiliations:** Department of Brain Sciences, Daegu Gyeongbuk Institute of Science and Technology (DGIST), Daegu, 42988 Republic of Korea

**Keywords:** G protein-coupled receptors, Permeation and transport, Molecular neuroscience

## Abstract

G protein-coupled receptors (GPCRs) regulate diverse intracellular signaling pathways through the activation of heterotrimeric G proteins. However, the effects of the sequential activation–deactivation cycle of G protein on the conformational changes of GPCRs remains unknown. By developing a Förster resonance energy transfer (FRET) tool for human M3 muscarinic receptor (hM3R), we find that a single-receptor FRET probe can display the consecutive structural conversion of a receptor by G protein cycle. Our results reveal that the G protein activation evokes a two-step change in the hM3R structure, including the fast step mediated by G_q_ protein binding and the subsequent slower step mediated by the physical separation of the Gα_q_ and Gβγ subunits. We also find that the separated Gα_q_-GTP forms a stable complex with the ligand-activated hM3R and phospholipase Cβ. In sum, the present study uncovers the real-time conformational dynamics of innate hM3R during the downstream G_q_ protein cycle.

## Abstract (web summary)

During G_q_ protein activation, the separated Gα_q_-GTP forms a stable complex with the ligand-activated hM3R and PLCβ. Here the authors demonstrate that a single M3 receptor FRET probe can display the real-time conformational dynamics of innate receptor by the downstream G_q_ protein cycle.

## Introduction

G protein-coupled receptors (GPCRs) are one of the largest families of membrane receptors, with more than 800 members encoded by about 3% of human genes^1–3^. They are widely expressed in cells organizing tissues and organs, and regulate cellular and physiological processes by transmitting various extracellular signals, including light, temperature, neurotransmitters, hormones, and odor substances, into cells^4,5^. The GPCR signals are transmitted to membrane-associated effectors like ion channels and enzymes through several heterotrimeric (Gαβγ) G proteins. As mediators, G proteins play an important role in enabling GPCR signaling to have high flexibility, sensitivity, and specificity^5^. For this reason, the way in which GPCRs and G proteins interact with each other to induce this signaling efficiency in living cells has been studied extensively^6–8^.

When GPCRs are activated by an agonist, the receptors combine with inactive heterotrimeric G proteins, followed by GDP release from the Gα subunit and GTP binding to the site, leading to the transient stabilization of the GPCR–G protein complexes^9^. Through biochemical and biophysiological methods, the interaction sites of these active-state GPCR–G protein complexes have been clarified in terms of the relationships between GPCR and Gα^10–12^, GPCR and Gβγ^13–16^, and Gα and Gβγ^10,17,18^. In more recent studies, high resolution structures of those complexes have been defined by cryogenic electron microscopy^19–25^.

Although they have been less studied compared with the active-state GPCR–G protein complexes, some resting-state complexes called preassembled inactive-state complexes have also been explored recently using ligand-binding and biochemical studies^24,26–28^ as well as biophysical studies in live cells^29–33^. These studies suggested that the preassembled complex formation may accelerate the onset of signaling by GPCRs and thus increase the GPCR sensitivity^33^. However, it has not yet been determined which subunit of heterotrimeric G proteins interacts with GPCRs in their preassembly or whether the preassembly is maintained during the receptor activation without further structural changes. Moreover, some studies have looked into the different receptor conformations during the different stages of the G protein activation cycle^34,35^, though the real-time effects of the G protein activation cycle on the innate conformation of GPCR remain unknown.

In this study, we develop a Förster resonance energy transfer (FRET) construct of human muscarinic acetylcholine receptor M3, hM3R-YFP-CFP, which enables the analysis of the conformational transition of hM3R that occurs during the coupled G_q_ protein cycling processes as well as the cascade from hM3R to phospholipase Cβ (PLCβ) within cells. Using this FRET construct, we find that the activation–deactivation cycle of G_q_ protein evokes multistep conformational changes of hM3R, which depend on the time-dependent association, separation, and dissociation of G_q_ protein subunits. We also provide evidence that the separated Gα_q_ and Gβγ subunits continuously stay on the agonist-activated hM3R, steadily activate PLCβ, and release from the receptor with different time constants. Our results demonstrate that the downstream G_q_ protein cycle inversely and dynamically regulates the upstream hM3R structure.

## Results

### A single hM3R-YFP-CFP FRET construct revealed a two-step hM3R activation signal

**Fig. 1:**
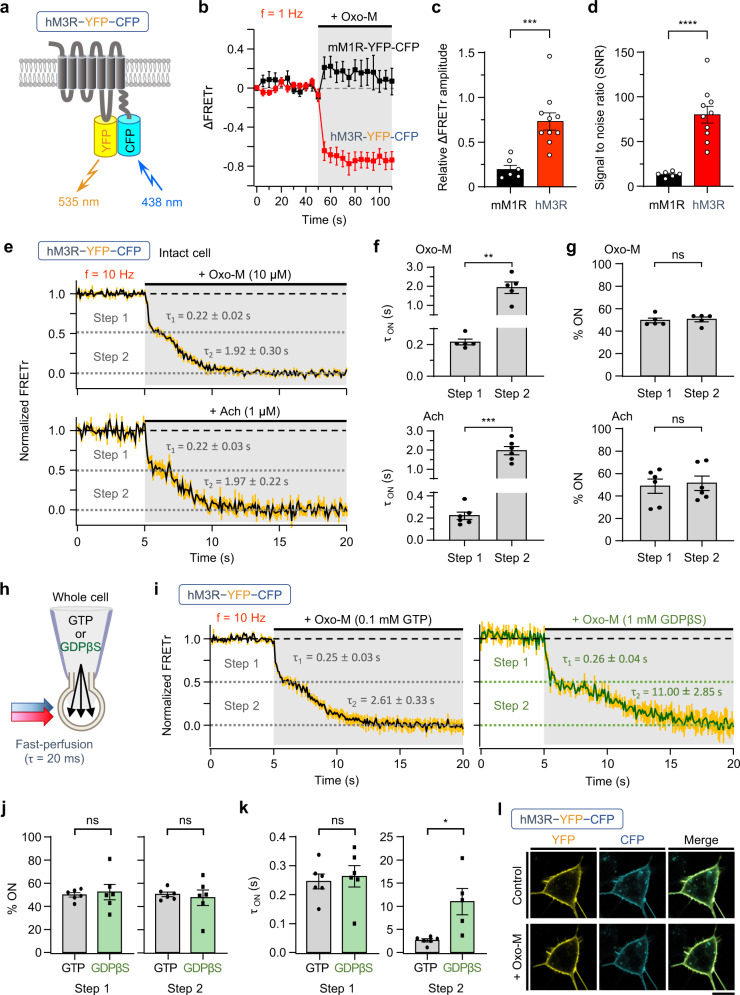
hM3R-YFP-CFP FRET construct shows a two-step activation. **a** Schematic of double-labeled hM3R construct hM3R-YFP-CFP at the plasma membrane. ∆FRETr signal (**b**), relative ∆FRETr amplitude (**c**) and SNR (**d**) in response to Oxo-M in cells expressing mM1R-YFP-CFP or hM3R-YFP-CFP. Sampling frequency: 1 Hz. mM3R, *n* = 6 (three cultures); hM3R, *n* = 10 (four cultures). Welch’s *t* test (two-sided, ***p = 0.0003, ****p < 0.0001). **e** Normalized FRETr signals in response to Oxo-M (top) or acetylcholine (Ach, bottom) in intact cell expressing hM3R-YFP-CFP. The FRETr changes from Oxo-M or Ach application show two-step activation decay in intact cells. Sampling frequency: 10 Hz. Yellow vertical lines indicate SEM. *n* = 5 (two cultures, top); *n* = 6 (two cultures, bottom). Time constant (*τ*_ON_) (**f**) and percent distribution (**g**) of each activation step in total FRETr response in intact cells treated with each agonist. Oxo-M, *n* = 5 (two cultures, Mann–Whitney test, two-sided, **p = 0.0079); Ach, *n* = 6 (two cultures, Welch’s *t* test, two-sided, ***p = 0.0004). **h** Whole-cell configuration of a cell with internal pipette solution containing 0.1 mM GTP or 1 mM GDPβS. **i** Two-step activation decay of FRETs by Oxo-M application in cells intracellularly perfused with 0.1 mM GTP (left) or 1 mM GDPβS (right). Sampling frequency: 10 Hz. Yellow vertical lines indicate SEM. GTP, *n* = 6 (three cultures); GDPβS, *n* = 6 (two cultures). Percent distribution (**j**) and *τ*_ON_ (**k**) of each step in the FRETr response under patched conditions. GTP, *n* = 6 (three cultures); GDPβS, *n* = 6 (two cultures). Welch’s *t* test (two-sided, *p = 0.0416). **l** Representative confocal images of a single cell expressing hM3R-YFP-CFP before (Control) and during Oxo-M application (+ Oxo-M). Experiments repeated independently for more than five cultures show similar results. Scale bar, 10 μm. Data are shown as mean ± SEM. ns not significant. Source data are provided as a Source Data file.

To investigate the signaling properties of M3R, a hM3R FRET construct, hM3R-YFP-CFP was constructed by replacing the third intracellular loop (ICL3) of wild-type hM3R with YFP and tagging CFP to the C-terminus (Fig. 1a) without damaging the selective recognition sites of G_q/11_-proteins (Supplementary Fig. 1a–c). The receptor was activated by applying the muscarinic receptor agonist oxotremorine-M (Oxo-M). In contrast to the previously reported mouse M1 muscarinic FRET probe (mM1R-YFP-CFP)^36^, binding of Oxo-M to this hM3R-YFP-CFP decreased the FRET ratio (FRETr) signal (Fig. 1b). In addition, hM3R-YFP-CFP showed ~3-fold larger FRETr responses and a ~5-fold higher signal-to-noise ratio (SNR) compared to mM1R-YFP-CFP (Fig. 1b–d). Photostability was also improved, and thus, the FRETr signal was maintained longer without a significant decrease compared to mM1R-YFP-CFP (Supplementary Fig. 1d, e). When the FRETr of the hM3R-YFP-CFP construct was measured in intact HEK293T cells under the faster perfusion system with higher frequency (*f* = 10 Hz), we found a two-step reduction of the FRETr signal with faster (step 1) and then slower (step 2) responses by Oxo-M treatment (Fig. 1e, top). There was a short delay (0.08 ± 0.05 s, *n* = 5) before the step 1. The time constant τ values of step 1 and step 2 were 0.22 ± 0.02 s and 1.92 ± 0.30 s (*n* = 5), respectively (Fig. 1f, top). However, the relative responsiveness of the two steps was almost equal at around 50% (Fig. 1g, top). Elevation of the sampling frequency to 100 Hz decreased the photostability but did not divide steps 1 and 2 further (Supplementary Fig. 1f). The FRETr change of the receptor was also measured after the application of the natural ligand acetylcholine (Ach). Consistent with the data of Oxo-M, 1 μM Ach induced two-step activation of hM3R-YFP-CFP with faster step 1 and slower step 2 responses (Fig. 1e–g, bottom). Both Oxo-M and Ach changed the FRETr signal in a concentration-dependent manner, but with significantly different EC_50_ values of 0.007 μM for Ach and 0.112 μM for Oxo-M (Supplementary Fig. 2). The results suggest that Ach had a higher affinity for hM3R-YFP-CFP receptor compared to Oxo-M, which was similar to the responses of other M3R FRET sensors^37^.

To characterize the two steps of the hM3R-YFP-CFP FRETr response, we manipulated intracellular GTP concentrations in the whole-cell configuration of the cells (Fig. 1h). In cells intracellularly perfused with the physiological level of GTP (0.1 mM), Oxo-M application induced almost the same two-step reduction in the FRETr signal of hM3R-YFP-CFP as those of intact cells (Fig. 1i, left). The cells patched with a pipette solution containing 1 mM of the GDP analog guanosine 5-O-(2-thiodiphosphate) (GDPβS) as a competitive antagonist of GTP binding to G proteins also displayed a two-step FRETr reduction with a similar proportion of step 1 and step 2 in the overall response (Fig. 1i, right, j). The time constant of the step 1 response was not changed, but the response of step 2 was nearly 5 times slower than that of cells with 0.1 mM of GTP (Fig. 1k). These results suggest that step 1 is likely related to the conformational change following the activation of hM3R-YFP-CFP by agonist binding, while step 2 represents the additional conformational changes of the receptor following the G protein activation. To check whether the internalization of the receptor affects the FRETr change, hM3R-YFP-CFP expression on the HEK293T cell membrane was visualized with a confocal microscope. We confirmed that there were no detectable changes in the fluorescence distribution of hM3R-YFP-CFP at the plasma membrane before and after the receptor activation (Fig. 1l).

To examine whether Oxo-M binding to hM3R-YFP-CFP normally evokes downstream intracellular signaling through the activation of coupled G_q_ protein, PLC-mediated hydrolysis of phosphatidylinositol 4,5-bisphosphate (PIP_2_) was measured in cells co-transfected with the PIP_2_ probe RFP-labeled pleckstrin homology domain of PLCδ_1_ (RFP-PH). Cells transfected with the wild-type mM1R or hM3R plasmid showed a strong translocation of RFP-PH from the plasma membrane to cytosol by Oxo-M treatment (Supplementary Fig. 3a–c). There was no significant movement of RFP-PH in cells expressing mM1R-YFP-CFP, confirming that mM1R-YFP-CFP cannot trigger downstream signaling pathways, as reported in a previous study^36^ (Supplementary Fig. 3d). However, Oxo-M induced strong translocation of RFP-PH in cells expressing hM3R-YFP-CFP, suggesting that the activation of hM3R-YFP-CFP by Oxo-M can stimulate downstream signaling pathways through G_q_ protein activation (Supplementary Fig. 3e). There was a slight difference in the relative RFP-PH changes at the plasma membrane and cytosol, but the half time of activation (*T*_50_) showed very similar kinetics to those of wild-type hM3R (Supplementary Fig. 3f, g). Those results were further confirmed in the experiments with Ach (Supplementary Fig. 4a, b). Though there was a considerable decrease in the relative fluorescence changes at the PM and cytosol (Supplementary Fig. 4c), the kinetics for the PIP_2_ hydrolysis were not changed in the FRET receptor (Supplementary Fig. 4d). Therefore, the results suggest that hM3R-YFP-CFP can transmit external signals to downstream pathways through the activation of the G_q_ protein with similar kinetics to that of wild-type hM3R.

### Two-step activation of hM3R-YFP-CFP can be explained by G_q_ protein activation

**Fig. 2:**
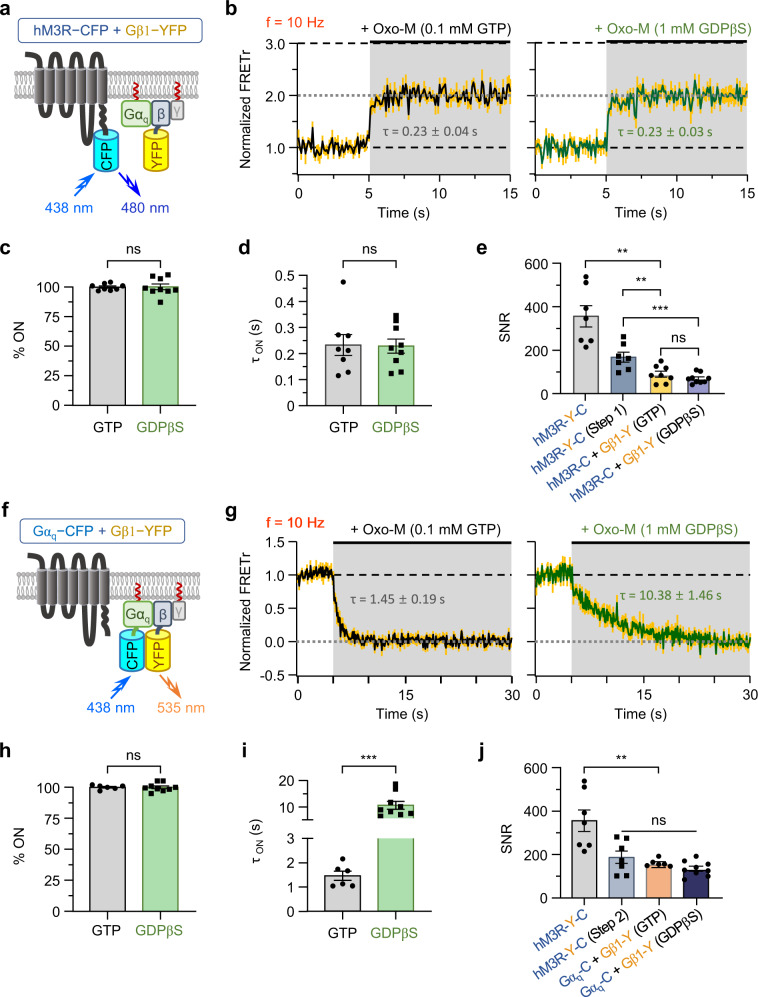
Two-step activation kinetics of hM3R-YFP-CFP FRET correspond to hM3R-G_q_ protein interaction and Gα_q_ and Gβγ dissociation, respectively. **a** Schematic of hM3R-CFP (hM3R-C), Gβ1-YFP (Gβ1-Y), and cognate G protein subunits at the plasma membrane. **b** Normalized FRETr in response to Oxo-M in cells intracellularly perfused with 0.1 mM GTP (left) or 1 mM GDPβS (right) through whole-cell configuration. Sampling frequency: 10 Hz. Yellow vertical lines indicate SEM. GTP, *n* = 8 (three cultures); GDPβS, *n* = 9 (three cultures). Percent of single step (% ON) in total FRETr response (**c**) and *τ*_ON_ of onset of FRETr change (**d**). GTP, *n* = 8 (three cultures); GDPβS *n* = 9 (three cultures). Welch’s t test (two-sided) and Student’s t test (two-sided), respectively. **e** SNR of ΔFRETr in response to Oxo-M in cells expressing each FRET sensor. The cells were intracellularly perfused with 0.1 mM GTP except the fourth column (1 mM GDPβS). First and second columns, *n* = 7 (three cultures); third column, *n* = 8 (four cultures); fourth column, *n* = 9 (three cultures). Welch’s *t* test (first-third columns), two-sided, **p = 0.0014; one-way ANOVA test with Tukey post hoc test (***p = 0.0003; second-third columns **p = 0.0043, second-fourth columns ***p = 0.0003, third-fourth columns *p* = 0.5728). **f** Schematic of wild-type hM3R, Gα_q_-CFP (Gα_q_-C), Gβ1-YFP, and Gγ2 at the plasma membrane. GRK2 was co-expressed but not presented in the depiction. **g** Normalized FRETr in response to Oxo-M in cells intracellularly perfused with 0.1 mM GTP (left) or 1 mM GDPβS (right) through whole-cell configuration. Sampling frequency: 10 Hz. GTP, *n* = 6 (two cultures); GDPβS, *n* = 9 (three cultures). Percent of single step in total FRETr response (**h**) and *τ*_ON_ of onset of FRETr change (**i**). GTP, *n* = 6 (two cultures); GDPβS, *n* = 9 (three cultures). Welch’s *t* test (two-sided, ***p = 0.0003). **j** SNR of ΔFRETr in response to Oxo-M application in cells expressing each FRET sensor. First and second columns, *n* = 7 (three cultures); third column, *n* = 6 (two cultures); fourth column, *n* = 9 (three cultures). Welch’s *t* test (first-third columns), two-sided, **p = 0.0074; one-way ANOVA test with Tukey post hoc test (*p* = 0.1299; second-third columns *p* = 0.5800, second-fourth columns *p* = 0.1099, third-fourth columns *p* = 0.6022). Data are shown as mean ± SEM. ns not significant. Source data are provided as a Source Data file.

Based on the similarity between hM3R-YFP-CFP and wild-type hM3R in terms of kinetics of PIP_2_ depletion, we further examined the molecular basis of the two steps. First, we hypothesized that the pathways corresponding to the fast step 1 of hM3R-YFP-CFP were related to a conformational change following Oxo-M binding to the receptor and subsequent interaction of the activated receptor with the heterotrimeric G_q_ protein. Thus, we performed FRET experiments in cells transfected with hM3R-CFP, Gβ1-YFP, and cognate G protein subunits (Fig. 2a). The results showed a single-step FRETr response composed of a fast signal upon Oxo-M treatment as reported in previous G_q_-coupled receptors^36,38^ (Fig. 2b, left, c, gray). The τ of the step (0.23 ± 0.04 s) (Fig. 2d, gray) was very similar to the step 1 τ of hM3R-YFP-CFP (Fig. 1k, left, gray), suggesting that the step 1 reaction includes fast coupling between hM3R and G_q_ protein after Oxo-M binding to the receptor. We conducted the same experiment yet 0.1 mM of GTP in the pipette solution was replaced with 1 mM of GDPβS to cross-check the similarity between the responses (Fig. 2b, right). The results showed that there was also a single FRETr signal response with almost the same time constant as that with 0.1 mM GTP (Fig. 2c, d, green) or step 1 reaction of hM3R-YFP-CFP (Fig. 1k, left), confirming that intracellular GTP concentration does not affect the step 1 reaction. We also measured the difference in the SNR of step 1 between hM3R-YFP-CFP and hM3R-CFP plus Gβ1-YFP (Fig. 2e). The SNR value (169 ± 23) of the step 1 response of hM3R-YFP-CFP was nearly twice those of hM3R-CFP plus Gβ1-YFP with 0.1 mM of GTP (90 ± 14) or with 1 mM of GDPβS (69 ± 8) (Fig. 2e; Supplementary Fig. 5a, b). There was no significant difference in the SNR between cells intracellularly perfused with GTP or GDPβS. Finally, Oxo-M induced the coupling of hM3R-CFP and Gβ1-YFP in a concentration-dependent manner with the EC_50_ value of ~2 μM (Supplementary Fig. 6a, c). When we measured the coupling between Gβ1-YFP and hM3R-BFP-CFP where the YFP was replaced by blue fluorescence protein (BFP), there was no significant change in the EC_50_ value (Supplementary Fig. 6b, c). The *τ*_ON_ value was not changed, but the SNR level decreased because of the partial reduction in the FRET signal amplitude (Supplementary Fig. 6d). These results suggest that the insertion of YFP to the ICL3 could attenuate the potency for the G_q_ protein coupling to our M3R FRET reporter.

We also performed FRET experiments between Gα_q_ and Gβγ subunits in cells expressing Gα_q_-CFP and Gβ1-YFP with wild-type hM3R, Gγ2, and GRK2 (Fig. 2f). GRK2 has been known to interact with G_q_ protein subunits^39,40^ to regulate the downstream signaling pathways. The results showed a single-step reduction of FRETr with 0.25 ± 0.08-s delay (*n* = 6) upon Oxo-M treatment (Fig. 2g, left, h, gray). The average *τ* value of this step was 1.45 ± 0.19 s (Fig. 2i gray), which was similar to that of the step 2 response of hM3R-YFP-CFP (Fig. 1k, right, gray). We conducted the same experiment in the presence of 1 mM of GDPβS instead of GTP (Fig. 2g, right). The results showed that with GDPβS, the response was much slower (10.38 ± 1.46 s) (Fig. 2i, green) compared to that of cells with GTP (Fig. 2i, gray) but similar to that of the step 2 response of hM3R-YFP-CFP with GDPβS (Fig. 1k, right, green). We also found that there was no significant difference in the SNR between hM3R-YFP-CFP (step 2) and Gα_q_-CFP plus Gβ1-YFP under the GTP or GDPβS condition (Fig. 2j; Supplementary Fig. 5c, d), suggesting that the step 2 FRET response of hM3R-YFP-CFP corresponds to the slow dissociation of Gα_q_ and Gβγ subunits after activation on the receptor. Using confocal microscopy we confirmed that the cells expressing hM3R-CFP plus Gβ1-YFP or Gα_q_-CFP plus Gβ1-YFP showed normal PIP_2_ hydrolysis through G_q_ protein activation (Supplementary Fig. 7a, c and 7b, d, bottom). Moreover, hM3R activation did not release any Gα_q_ and Gβγ subunits from the plasma membrane to the cytosol (Supplementary Fig. 7a, c and 7b, d, top), suggesting that both subunits were independently tethered to the plasma membrane after separation.

### Oxo-M dissociation evoked two-step deactivation kinetics in hM3R-YFP-CFP

We also measured the deactivation kinetics of hM3R-YFP-CFP after Oxo-M washout. As shown in Supplementary Fig. 8a, hM3R-YFP-CFP deactivation showed a two-step FRETr recovery with faster step 1 and slower step 2 reactions. To determine the nature of these two steps, FRETr was also measured during the receptor deactivation in cells expressing hM3R-CFP plus Gβ1-YFP or Gα_q_-CFP plus Gβ1-YFP (Supplementary Fig. 8b, c). As a result, the deactivations were observed as a single step in both experiments. The deactivation τ of hM3R-CFP plus Gβ1-YFP (0.53 ± 0.04 s) showed a value very similar to that of step 1 (0.47 ± 0.08 s) of hM3R-YFP-CFP deactivation (Supplementary Fig. 8d), whereas that of Gα_q_-CFP plus Gβ1-YFP (21.37 ± 1.34 s) was similar to that of step 2 (20.02 ± 2.29 s) of hM3R-YFP-CFP deactivation (Supplementary Fig. 8e). In terms of percent distribution of each step in the total recovery, both hM3R-CFP plus Gβ1-YFP and Gα_q_-CFP plus Gβ1-YFP showed a similar distribution value of around 88% (Supplementary Fig. 8f, g). We further confirmed that, consistent with previous findings^36^, the coexpression of GRK2 significantly increased the signal amplitude and SNR of the FRETr response between Gα_q_-CFP and Gβ1-YFP but did not change the kinetics of receptor-induced activation or deactivation (Supplementary Fig. 9). GRK2 expression neither affect the kinetics nor the peak responses of FRETr in hM3R-YFP-CFP, but decreased the SNR by slightly enhancing the noise level (Supplementary Fig. 10).

### hM3R-YFP-CFP can make a complex with the separated Gβγ subunits

**Fig. 3:**
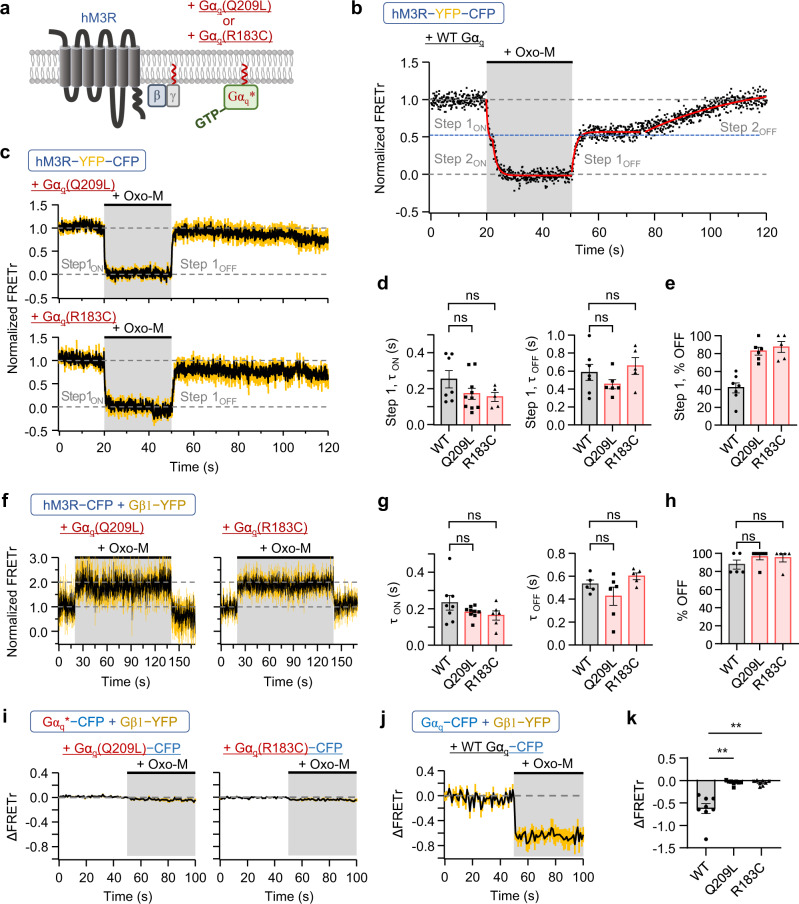
hM3R-YFP-CFP shows only step 1_ON_ and 1_OFF_ in presence of constitutively active Gα_q_ proteins. **a** Schematic of wild-type (WT) hM3R with the constitutively active mutant form of Gα_q_, Gα_q_(Q209L) or Gα_q_(R183C) at the plasma membrane. **b** Normalized FRETr signal in response to Oxo-M in cell expressing hM3R-YFP-CFP and WT G_q_ proteins (Gα_q_, Gβ1, and Gγ2). Sampling frequency: 10 Hz. Red lines are single-exponential fits for step 1_ON_, step 2_ON_, step 1_OFF_, and step 2_OFF_. **c** Normalized FRETr in cells expressing hM3R-YFP-CFP, mutant Gα_q_ (Q209L or R183C), and cognate G protein subunits. Sampling frequency: 10 Hz. Yellow vertical lines indicate SEM. Q209L, *n* = 6 (two cultures); R183C, *n* = 5 (two cultures). **d** *τ* for step 1_ON_ and 1_OFF_. For τ_ON_, WT, *n* = 7 (three cultures); Q209L, *n* = 10 (two cultures); R183C, *n* = 5 (two cultures). One-way ANOVA test (*p* = 0.1972) with Tukey post hoc test (WT-Q209L, *p* = 0.2688; WT-R183C, *p* = 0.2497; Q209L-R183C, *p* = 0.9445). For *τ*_OFF_, WT, *n* = 7 (three cultures); Q209L, *n* = 6 (two cultures); R183C, *n* = 5 (two cultures). One-way ANOVA test (p = 0.2522) with Tukey post hoc test (WT-Q209L, *p* = 0.4861; WT-R183C, *p* = 0.8014; Q209L-R183C, *p* = 0.2390). **e** FRETr recovery (% OFF) of step 1. WT, *n* = 7 (three cultures); Q209L, *n* = 6 (two cultures); R183C, *n* = 5 (two cultures). **f** Normalized FRETr signals in cells expressing hM3R-CFP, Gβ1-YFP, mutant Gα_q_ (Q209L or R183C), and Gγ2. Sampling frequency: 10 Hz. Q209L, *n* = 6 (two cultures); R183C, *n* = 5 (two cultures). **g** τ of onset and offset of FRETr changes by Oxo-M treatment. For τ_ON_, WT, *n* = 8 (two cultures); Q209L, *n* = 9 (two cultures); R183C, *n* = 6 (two cultures). One-way ANOVA test (*p* = 0.2202) with Tukey post hoc test (WT-Q209L, *p* = 0.3672; WT- R183C, *p* = 0.2369; Q209L-R183C, *p* = 0.8971). For *τ*_OFF_, WT, *n* = 5 (two cultures); Q209L, *n* = 6 (two cultures); R183C, *n* = 5 (two cultures). One-way ANOVA test (*p* = 0.1270) with Tukey post hoc test (WT-Q209L, *p* = 0.4149; WT-R183C, *p* = 0.6929; Q209L-R183C, *p* = 0.1134). **h** FRETr recovery (% OFF). WT, *n* = 5 (two cultures); Q209L, *n* = 6 (two cultures); R183C, *n* = 5 (two cultures). One-way ANOVA test (*p* = 0.3380) with Tukey post hoc test (WT-Q209L, *p* = 0.3479; WT-R183C, *p* = 0.4737; Q209L-R183C, *p* = 0.9800). ΔFRETr signals in cells expressing WT hM3R, Gα_q_-CFP (Q209L or R183C (**i**), WT (**j**))_,_ Gβ1-YFP, Gγ2, and GRK2. Sampling frequency: 1 Hz. Yellow vertical lines indicate SEM. Q209L, *n* = 8 (two cultures); R183C, *n* = 8 (two cultures); WT Gα_q_, *n* = 8 (two cultures). **k** Average ΔFRETr during Oxo-M application in experiments (**i**) and (**j**). Welch’s ANOVA test (*p* = 0.0008) with Games–Howell post hoc test (WT-Q209L, **p = 0.0025; WT-R183C, **p = 0.0026; Q209L-R183C, *p* = 0.9925). Data are shown as mean ± SEM. ns not significant. Source data are provided as a Source Data file.

To further elucidate how hM3R communicates with G_q_ proteins to transmit external signals to the downstream pathways, we constructed two constitutively active Gα_q_ mutant forms: Gα_q_(Q209L) and Gα_q_(R183C) (Fig. 3a). Previous studies have reported that diseases such as uveal melanoma result from continuous GPCR activity through Gα_q_ mutation (Q209L or R183C) and loss of GTPase activity of the subunit^41–43^. First, we examined if the additional expression of wild-type G_q_ proteins affects the Oxo-M-induced FRETr signal of hM3R-YFP-CFP (Supplementary Fig. 11). As shown in Fig. 3b and Supplementary Fig. 12, the overexpression of wild-type G_q_ proteins did not change the two-step kinetics for activation and deactivation, the percentage recovered, or the SNR of hM3R-YFP-CFP. However, in cells co-transfected with mutant Gα_q_(Q209L or R183C) and cognate G protein subunits, only a single step of activation and recovery was identified in the FRETr experiment (Fig. 3c), showing a comparable time constant to step 1 (ON or OFF) of wild-type G_q_ protein (Fig. 3d). Because of the absence of step 2, the FRETr was almost completely recovered within a single step (Fig. 3e). Consistently, after the intracellular perfusion of the nonhydrolyzable GTP analog guanosine 5-O-(3-thiotriphosphate) (GTPγS), Oxo-M evoked a single-step activation of FRETr probably because GTPγS activated the G proteins almost irreversibly^44^ (Supplementary Fig. 13).

Next, we performed the FRETr experiment between hM3R-CFP and Gβ1-YFP in cells co-expressing mutant Gα_q_(Q209L or R183C) and Gγ2. The results showed that Oxo-M induced a single-step FRETr change with similar time constants and percentage recovered, but significantly lower SNR compared to those of wild-type Gα_q_ (Fig. 3f–h; Supplementary Fig. 14). These results suggest that, upon Oxo-M binding to the receptor, hM3R can independently interact with the separated Gβγ subunits though its binding affinity is weaker than that for heterotrimeric G protein. Interestingly, the results also showed that the interaction between the hM3R and Gβγ subunit was sustained during the application of the agonist and recovered immediately after Oxo-M washout. Finally, a FRETr experiment was performed in cells expressing hM3R, mutant Gα_q_(Q209L or R183C)-CFP, Gβ1-YFP, Gγ2, and GRK2. The results showed no changes in FRETr by Oxo-M application, except in wild-type Gα_q_ (Fig. 3i–k), indicating that the mutant Gα_q_(Q209L or R183C) and Gβγ subunits were dissociated in the resting condition and thus responded to receptor activation separately.

### Inhibition of GDP release from Gα_q_ selectively blocked the step 2 response of hM3R-YFP-CFP

**Fig. 4:**
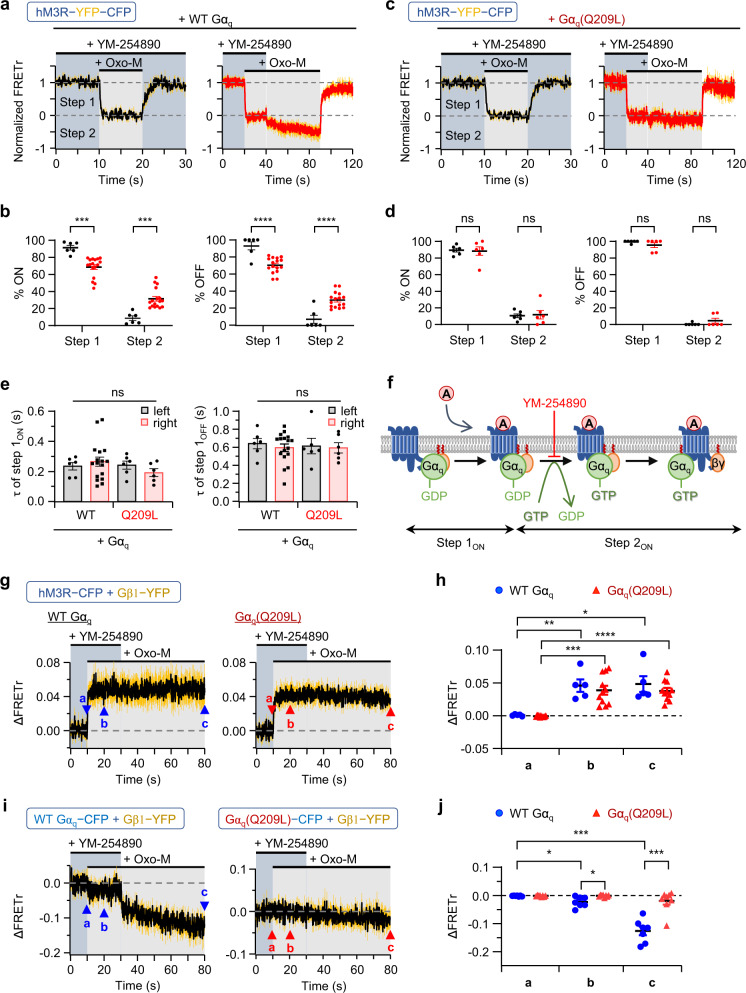
YM-254890 (YM) blocks the step 2 stage of hM3R-YFP-CFP activation. In figures (**a–e**), cells were transfected with hM3R-YFP-CFP, Gα_q_ (wild-type (WT) or Q209L), and cognate G protein subunits. Time courses of normalized FRETr signals in response to Oxo-M in cells expressing WT Gα_q_ (**a**) or mutant Gα_q_ (Q209L) (**c**) during continuous (left black trace, *n* = 6, two cultures, respectively) or temporary (right red trace, *n* = 17, three cultures for WT; *n* = 6, two cultures for Gα_q_ (Q209L)) medication of YM. Sampling frequency: 10 Hz. Yellow vertical lines indicate SEM of each time point. **b** Percent distribution of each step during activation (ON) or deactivation (OFF) of hM3R-YFP-CFP in figure (**a**). Student’s *t* test (% ON, two-sided, ***p = 0.0002; % OFF, two-sided, ****p < 0.0001). **d** Percent distribution of each step during hM3R-YFP-CFP activation (ON) or deactivation (OFF) in figure (**c**). Student’s *t* test (% ON, two-sided) and Welch’s t test (% OFF, two-sided). **e** *τ* of step 1_ON_ and step 1_OFF_ shown in figures (**a**) and (**c**). The values of step 2 were not compared. Two-way ANOVA test with Tukey post hoc test. **f** Schematic showing the action step of YM. **g** Time course of ∆FRETr signals in response to Oxo-M in cells expressing hM3R-CFP, Gβ1-YFP, Gγ2, and WT Gα_q_ (left, *n* = 5, two cultures) or mutant Gα_q_(Q209L) (right, *n* = 11, three cultures) during temporary medication of YM. Sampling frequency: 10 Hz. **h** Average of ∆FRETr at each marked time period (a: 0.0–9.9 s; b: 10–20 s; c: 70–80 s) in figure (**g**). Paired *t* test (two-sided). WT Gα_q_ (**a**, **b** **p = 0.0077; **a**–**c** *p = 0.0163), Gα_q_(Q209L) (**a**, **b** ***p = 0.0002; **a**–**c** ****p < 0.0001). **i** Time course of ∆FRETr signals in response to Oxo-M in cells expressing hM3R, Gβ1-YFP, Gγ2, GRK2, and WT Gα_q_-CFP (left, *n* = 7, two cultures) or mutant Gα_q_(Q209L)-CFP (right, *n* = 7, two cultures) during temporary medication of YM. **j** Average of ∆FRETr at each marked time period in (**i**). Paired *t* test (two-sided) within WT Gα_q_ (**a**–**b** *p = 0.0176; **a**–**c** ***p = 0.0002), and Welch’s *t* test (two-sided) between WT Gα_q_ and Gα_q_(Q209L) at each time point (**b** *p = 0.0197; **c** ***p = 0.0002). Data are shown as mean ± SEM. ns not significant. Source data are provided as a Source Data file.

YM-254890 (YM) has been studied as a candidate drug for diverse diseases, such as thrombosis, asthma, and melanoma^43^. Preclinical studies have shown that YM’s main mechanism of action is the inhibition of GDP release from the Gα_q_ protein^43^. We examined the effects of YM on the FRETr response of the hM3R and G_q_ proteins. As seen in Fig. 4a, left, in the presence of YM, only the step 1 (ON and OFF) FRETr response was detected upon Oxo-M application in cells expressing hM3R-YFP-CFP and wild-type G_q_ proteins. When the YM was removed in the middle of hM3R-YFP-CFP activation with Oxo-M, the cells showed a slow step 2_ON_ FRETr response up to about 31% of the total FRETr activation (step 1_ON_ + step 2_ON_) and a step 2_OFF_ response up to about 30% of the total deactivation (step 1_OFF_ + step 2_OFF_) (Fig. 4a, b, red). However, the results of experiments with mutant Gα_q_(Q209L) revealed that there was a normal step 1 FRETr response by Oxo-M but that the step 2 response was not detected in hM3R-YFP-CFP even after washing out YM (Fig. 4c, d). This could have been due to the pre-separation of constitutively active mutant Gα_q_ and βγ subunits before Oxo-M treatment. There was no significant difference in the τ values of step 1 (ON and OFF) between wild-type G_q_ and G_q_(Q209L) (Fig. 4e). These results suggest that YM did not affect the interaction of G_q_ protein with Oxo-M-activated hM3R but selectively inhibited the step 2 response by blocking the reaction of GDP release from the Gα_q_ protein and the following GTP binding and separation of the heterotrimeric G_q_ protein to Gα_q_ and βγ subunits (Fig. 4f).

The molecular mechanism of the single-step FRET response under YM treatment was further studied in cells expressing hM3R-CFP plus Gβ1-YFP. Consistently, as shown in Fig. 4g, h, YM did not affect the interaction between hM3R-CFP and Gβ1-YFP by Oxo-M. Moreover, the interaction between hM3R-CFP and Gβ1-YFP in cells expressing mutant G_q_(Q209L) was normal. We also examined the effects of YM in cells expressing Gα_q_-CFP plus Gβ1-YFP. In those cells, YM inhibited the activation of the G_q_ protein and thus blocked the separation of Gα_q_-CFP and Gβ1-YFP (Fig. 4i, left). Then, after the removal of the YM, there was a slow FRETr decrease by the separation of Gα_q_ and βγ subunits. Interestingly, a minor but significant decrease in FRETr was detected after Oxo-M application even in the presence of YM, suggesting that the association of heterotrimeric G_q_ protein with ligand-activated hM3R may result in the structural rearrangement between Gα_q_ and Gβγ subunits although they are still closely associated with each other. In cells expressing mutant Gα_q_(Q209L)-CFP plus Gβ1-YFP, there were no changes in FRETr after YM washout, supporting the idea that Gα_q_(Q209L)-CFP and Gβ1-YFP were already separated and not affected by either YM or receptor activation (Fig. 4i, j). We additionally examined whether this Gα_q_(Q209L)-CFP, which has no interaction with Gβγ, is normally anchored to the plasma membrane. The confocal microscopy imaging showed no difference in the fluorescent expression level of Gα_q_-CFP and Gβ1-YFP in the plasma membrane or cytosol depending on whether Gα_q_ was mutated in the resting state (Supplementary Fig. 15a–f). Moreover, in both conditions, receptor activation did not change those signal levels (Supplementary Fig. 15a–d). However, there was a difference in the regulation of PIP_2_ levels. Oxo-M hydrolyzed the PIP_2_ and increased the intracellular level of RFP-PH in cells expressing wild-type Gα_q_-CFP and Gβ1-YFP (Supplementary Fig. 15a). In contrast, in cells expressing mutant Gα_q_(Q209L)-CFP and Gβ1-YFP, the cytosolic level of RFP-PH was already elevated regardless of receptor activation, confirming that Gα_q_(Q209L)-CFP can activate PLC even without receptor stimulation (Supplementary Fig. 15b).

### Two-step structural change in hM3R-YFP-CFP depends on G protein coupling

**Fig. 5:**
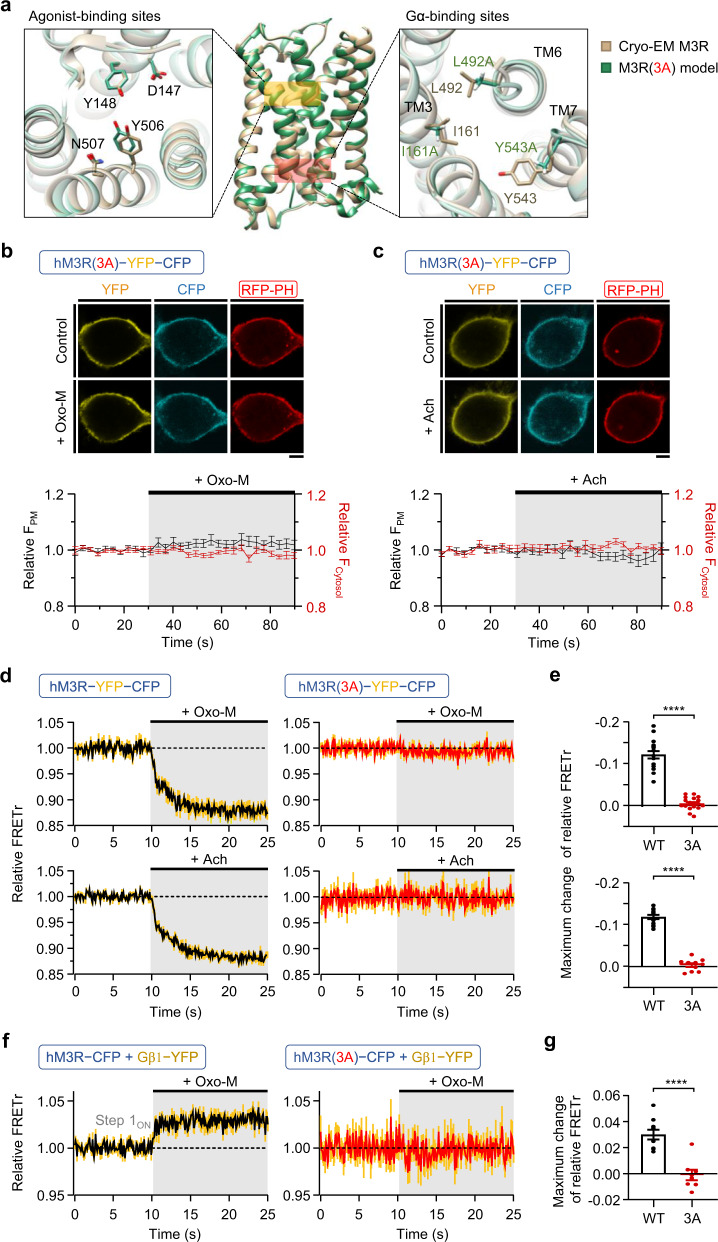
hM3R(3A) is not able to bind G_q_ protein. **a** Comparison of cryo-EM M3R (PDB ID: 4DAJ)^53^ and I-TASSER 3D predicted M3R(3A) on orthosteric agonist-binding sites (left) and Gα-binding sites (right, 3x46 (I161), 6x37 (L492), 7x53 (Y543)). The yellow and red boxes in the middle panel indicate agonist- and Gα-binding regions of M3R, respectively. Amino acid residues on binding sites are shown as a stick in tan (cryo-EM M3R) or green (M3R(3A) model). Top, representative confocal images of cells expressing red fluorescent protein-labeled pleckstrin homology domain of PLCδ_1_ (RFP-PH) and hM3R(3A)-YFP-CFP were obtained before (Control) and during 10 μM Oxo-M (**b**) or Ach (**c**) application. Scale bar, 5 µm. Bottom, time courses of relative fluorescence intensity of RFP-PH at plasma membrane (black trace, left axis) and cytosol (red trace, right axis) were measured in 10-11 cells from three independent experiments. Sampling frequency: 0.33 Hz. **d** Time course of relative FRETr in response to 15 s of 10 μM Oxo-M (top) or Ach (bottom) in cells expressing wild-type (WT) hM3R-YFP-CFP (left) or hM3R(3A)-YFP-CFP (right). Sampling frequency: 10 Hz. Oxo-M treated hM3R-YFP-CFP, *n* = 16 (two cultures); Oxo-M treated hM3R(3A)-YFP-CFP, *n* = 18 (two cultures); Ach treated hM3R-YFP-CFP, *n* = 12 (three cultures); Ach treated hM3R(3A)-YFP-CFP, *n* = 10 (two cultures). **e** Maximum change of relative FRETr in each cell of figure (**d**) by 10 μM Oxo-M (top) or Ach (bottom) treatment. Welch’s *t* test (top) (two-sided, ****p < 0.0001), and Student’s *t* test (bottom) (two-sided, ****p < 0.0001). **f** Time course of relative FRETr in response to 15 s of Oxo-M in cells expressing Gα_q_, Gβ1-YFP, Gγ2, and hM3R-CFP (left, *n* = 10, two cultures) or hM3R(3A)-CFP (right, *n* = 8, three cultures). Sampling frequency: 10 Hz. Yellow vertical lines indicate SEM. **g** Maximum change of relative FRETr in each cell of (**f**) by Oxo-M treatment. Student’s *t* test (two-sided, ****p < 0.0001). Data are shown as mean ± SEM, with error bars indicating SEM. Source data are provided as a Source Data file.

Existing fluorescence-based M3 sensors commonly show a single-step FRET change, which represents the conformational activation of a receptor by agonist binding^37,38,45–47^. To examine whether the two-step FRET signal observed in hM3R-YFP-CFP, especially in step 1, involves receptor activation in addition to G_q_-protein coupling, we applied a class A GPCR mutation strategy that blocks Gα binding to our hM3R-YFP-CFP construct without impairing receptor activation by the ligand^48^. The three amino acid residues at positions 3x46 in TM3, 6x37 in TM6, and 7x53 in TM7 (notation represents the GPCRdb numbering scheme^49^ from GPCRdb [http://www.gpcrdb.org]^50^) are conserved in all class A GPCRs and important for the cytosolic release of 6x37, which comes in contact with a universally conserved leucine residue (position G.H5.25; notation taken from “The Common G Protein Numbering Scheme”^51^) of the Gα subunit^48^ upon activation. This leucine residue plays a pivotal role in the coupling of Gα to GPCRs^52^. Through modeling of the 3D structure of M3R(3A) using both I-TASSER and SWISS-Model, we confirmed that the allosteric effect of alanine substitution on the structure or operation of the receptor’s agonist-binding sites^53^ was insignificant^48^ (Fig. 5a, left; Supplementary Fig. 16, left). To block the G_q_ protein coupling of hM3R-YFP-CFP, we substituted the corresponding I162 (3x46), L493 (6x37), and Y544 (7x53) residues with alanine in the construct. We found that the mutant hM3R(3A)-YFP-CFP was normally expressed in the cell membrane, but PIP_2_ was not hydrolyzed even after the receptor was activated by Oxo-M or Ach (Fig. 5b, c). Next, the FRET signal of hM3R(3A)-YFP-CFP was measured after the treatment with these two agonists. Unlike the wild-type hM3R-YFP-CFP, hM3R(3A)-YFP-CFP displayed no considerable FRETr changes (Fig. 5d, e). These results suggested that hM3R(3A)-YFP-CFP could not interact with the heterotrimeric G_q_ protein; this result was consistent with previous findings on class A receptors^48^. We also conducted FRET experiments between hM3R(3A)-CFP and Gβ1-YFP. As expected, the 3A mutant receptor did not interact with G_q_ proteins (Fig. 5f, g). Therefore, the FRETr signal of hM3R-YFP-CFP represents the conformational changes induced by G_q_ protein coupling but not the structural activation of the receptor by ligand binding. To convincingly show that the step 1 of hM3R-YFP-CFP is caused by G_q_ coupling and not by ligand binding-induced conformational changes, we also examined the activity of single-leucine mutant (3x50) M3R which has been shown to block G_q_ signaling but still undergo ligand-induced conformational rearrangement and arrestin-coupling^54^. As a result, the mutant hM3R(R166L^3x50^)-YFP-CFP displayed no considerable PIP_2_ hydrolysis and FRETr changes (Supplementary Fig. 17).

### Independent binding of Gβγ to hM3R may require the coupling of Gα_q_-GTP to the receptor

**Fig. 6:**
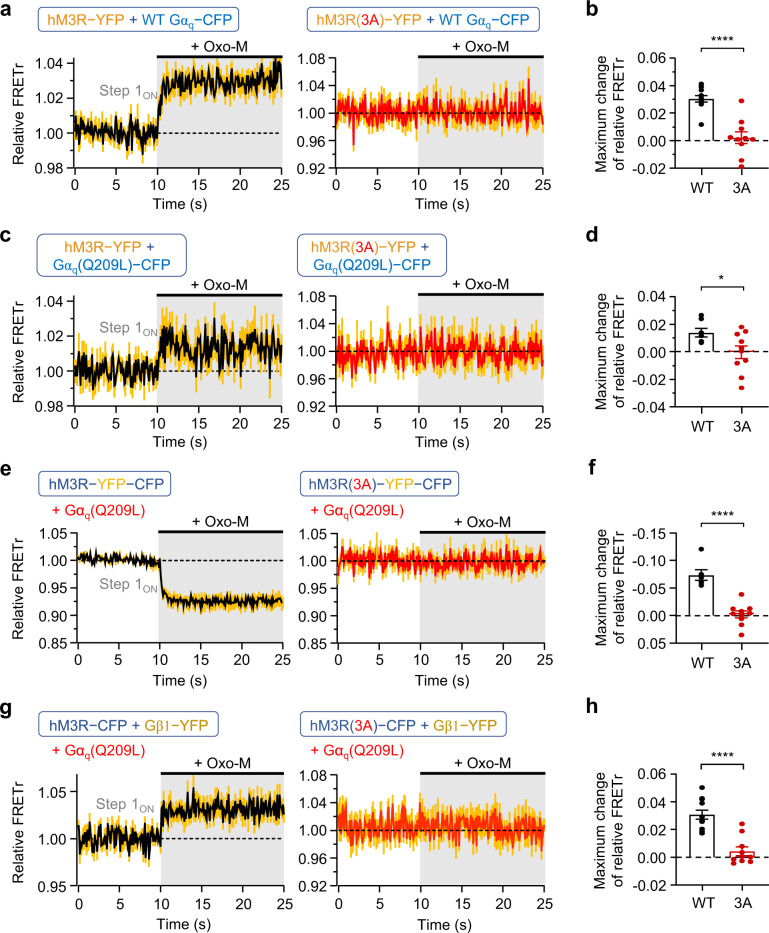
hM3R(3A) does not interact with Gβγ subunits independently. **a** Time course of relative FRETr in response to 15 s of Oxo-M in cells expressing Gα_q_-CFP, Gβ1, Gγ2, and hM3R-YFP (left, *n* = 11, two cultures) or hM3R(3A)-YFP (right, *n* = 10, two cultures). Sampling frequency: 10 Hz. Yellow vertical lines indicate SEM. **b** Maximum change of relative FRETr in cells expressing each FRET sensor by Oxo-M treatment. WT, *n* = 11 (two cultures); 3A, *n* = 10 (two cultures). Student’s *t* test (two-sided, ****p < 0.0001). **c** Time course of relative FRETr in response to 15 s of Oxo-M in cells expressing Gα_q_(Q209L)-CFP, Gβ1, Gγ2, and hM3R-YFP (left, *n* = 7, two cultures) or hM3R(3A)-YFP (right, *n* = 10, two cultures). Sampling frequency: 10 Hz. **d** Maximum change of relative FRETr in cells expressing each FRET sensor by Oxo-M treatment. WT, *n* = 7 (two cultures); 3A, *n* = 10 (two cultures). Student’s *t* test (two-sided, *p = 0.0352). **e** Time course of relative FRETr in response to 15 s of Oxo-M in cells expressing Gα_q_(Q209L), Gβ1, Gγ2, and hM3R-YFP-CFP (left, *n* = 6, two cultures) or hM3R(3A)-YFP-CFP (right, *n* = 10, two cultures). Sampling frequency: 10 Hz. **f** Maximum change of relative FRETr in cells expressing each FRET sensor by Oxo-M treatment. WT, *n* = 6 (two cultures); 3A, *n* = 10 (two cultures). Student’s *t* test (two-sided, ****p < 0.0001). **g** Time course of relative FRETr in response to 15 s of Oxo-M in cells expressing Gα_q_(Q209L), Gβ1-YFP, Gγ2, and hM3R-CFP (left, *n* = 11, two cultures) or hM3R(3A)-CFP (right, *n* = 10, two cultures). Sampling frequency: 10 Hz. **h** Maximum change of relative FRETr in cells expressing each FRET sensor by Oxo-M treatment. WT, *n* = 11 (two cultures); 3A, *n* = 10 (two cultures). Student’s *t* test (two-sided, ****p < 0.0001). Data are shown as mean ± SEM, with error bars indicating SEM. Source data are provided as a Source Data file.

To further understand the coupling mechanism between hM3R and separated Gβγ subunits, we examined the independent Gβγ interaction with the mutant hM3R(3A). First, consistent with the results shown in Fig. 5f, g, the findings indicated that coupling was normal between the wild-type hM3R-YFP and Gα_q_-CFP but completely abolished between hM3R(3A)-YFP and Gα_q_-CFP (Fig. 6a, b). Similarly, Gα_q_(Q209L)-CFP coupled with the wild-type hM3R-YFP but not with hM3R(3A)-YFP (Fig. 6c, d). Interestingly, the maximum change in the relative FRETr signal was strongly reduced in cells expressing the wild-type hM3R-YFP plus Gα_q_(Q209L)-CFP compared with that in cells expressing the wild-type hM3R-YFP plus wild-type Gα_q_-CFP (WT Gα_q_-CFP: 0.030 ± 0.002; Gα_q_(Q209L)-CFP: 0.014 ± 0.003, ***p = 0.0006; Fig. 6b, d). Next, we measured the FRETr signal in cells expressing hM3R(3A)-YFP-CFP, Gα_q_(Q209L), and the rest of the wild-type cognate G protein subunits. Unlike the responses of the wild-type hM3R-YFP-CFP (Fig. 6e, f, left), the FRETr signal did not decrease in hM3R(3A)-YFP-CFP (Fig. 6e, f, right). Finally, we examined the direct binding of the separated Gβγ to hM3R in cells co-transfected with Gα_q_(Q209L). As shown in Fig. 6g, h, FRETr did not change in cells expressing the mutant hM3R(3A)-CFP. These results suggest that the separated Gβγ subunits could bind only to receptors coupled with Gα_q_-GTP.

### Sustained assembly of hM3R, Gα_q_, and PLCβ_1_ during receptor activation

**Fig. 7:**
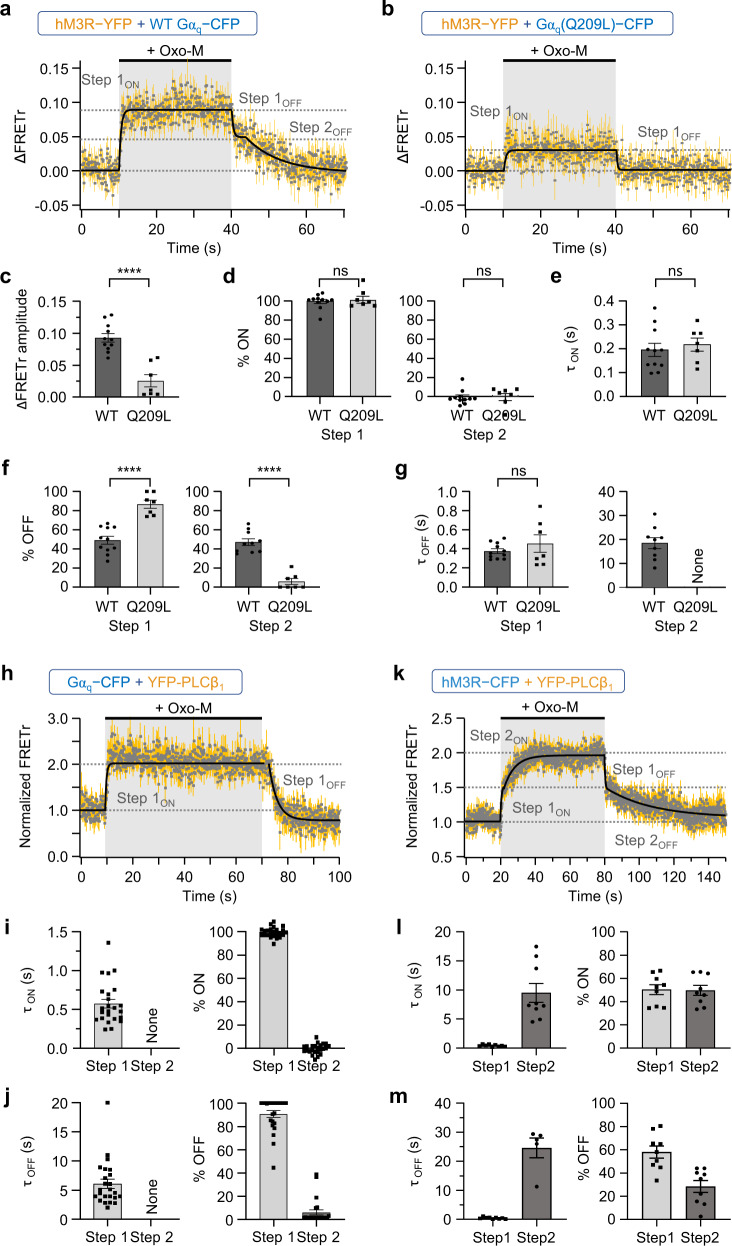
hM3R, Gα_q_, and PLC form a heterotrimer during receptor activation. Time course of ∆FRETr in response to 30 s of Oxo-M in cells expressing hM3R-YFP, Gβ1, Gγ2 and wild-type (WT) Gα_q_-CFP (**a**) or Gα_q_(Q209L)-CFP (**b**). Sampling frequency: 10 Hz. Yellow vertical lines indicate SEM. WT, *n* = 11 (two cultures); Q209L, *n* = 7 (two cultures). **c** Amplitude of ∆FRETr in cells expressing each FRET sensor. WT, *n* = 11 (two cultures); Q209L, *n* = 7 (two cultures). Student’s *t* test (two-sided, ****p < 0.0001). **d** Percent distribution of each step in total FRETr response. WT, *n* = 11 (two cultures); Q209L, *n* = 7 (two cultures). Student’s *t* test (two-sided). **e** *τ*_ON_ of step 1_ON_. WT, *n* = 11 (two cultures); Q209L, *n* = 7 (two cultures). Student’s *t* test (two-sided). **f** Percent of each decreasing step in contrast to step 1_ON_. WT, *n* = 11 (two cultures); Q209L, *n* = 7 (two cultures). Student’s *t* test (two-sided, ****p < 0.0001). **g** *τ*_OFF_ of each deactivation step. WT, *n* = 11 (two cultures); Q209L, *n* = 7 (two cultures). Welch’s *t* test (two-sided). **h**, **k** Time course of normalized FRETr in response to 60 s of Oxo-M in cells expressing hM3R, Gα_q_-CFP, Gβ1, Gγ2, and YFP-PLCβ_1_ (**h**, *n* = 24, two cultures) or in cells expressing hM3R-CFP, and YFP-PLCβ_1_ (**k**, *n* = 9, two cultures). Sampling frequency: 10 Hz. Yellow vertical lines indicate SEM. **i**, **l** *τ*_ON_ of each activation step and percent distribution of each step in total FRETr response (**i**, *n* = 24, two cultures; **l**, *n* = 9, two cultures). **j**, **m** *τ*_OFF_ of each deactivation step and percent of each decreasing step in contrast to total FRETr response of activation (**j**, *n* = 24, two cultures; **m**, *n* = 9, two cultures). Data are shown as mean ± SEM, with error bars indicating SEM. ns not significant. Source data are provided as a Source Data file.

To determine the operating role of Gα_q_ in the regulation of hM3R conformation, we performed FRETr experiments in cells transfected with hM3R-YFP, Gα_q_-CFP, and cognate G protein subunits. The receptor activation upon Oxo-M application showed a single-step FRETr increase, suggesting the interaction of hM3R-YFP with Gα_q_-CFP (Fig. 7a, d). Interestingly, the increased FRETr signal was maintained during the Oxo-M treatment. After Oxo-M removal, the FRETr signal returned to baseline through two-step kinetics with almost equal proportions (Fig. 7a, f). To investigate this two-step recovery, we first conducted the same FRETr experiments in the presence of YM (Supplementary Fig. 18a). There was a single-step increase in FRETr like without YM, with a τ of around 0.2 s (Fig. 7e; Supplementary Fig. 18f, left). This was very similar to the step 1 τ (~0.21 s) of hM3R-YFP-CFP, confirming that the fast step 1_ON_ response of hM3R-YFP-CFP is mediated by the coupling of hM3R with heterotrimeric inactive G_q_ protein. Importantly, in the presence of YM, there was only a single-step FRETr recovery (Supplementary Fig. 18a), with the time constant similar to the step 1_OFF_ of hM3R-YFP plus Gα_q_-CFP (Fig. 7g) and step 1_OFF_ of hM3R-YFP-CFP (Supplementary Fig. 8a, d). Based on these results, we can speculate that the two-step recovery of FRETr in cells with hM3R-YFP and Gα_q_-CFP can be defined as the step 1_OFF_ and step 2_OFF_ responses in hM3R-YFP-CFP, where step 1_OFF_ may indicate the release of Gβγ subunit from hM3R after the uncoupling of Oxo-M from hM3R and step 2_OFF_ could be a result of the dissociation of Gα_q_-CFP from hM3R-YFP after the hydrolysis of GTP to GDP by the GTPase activity of Gα_q_ (Fig. 7a). This speculation was further tested using the mutant Gα_q_(Q209L). As shown in Fig. 7b, f, no significant step 2_OFF_ response was observed after the Oxo-M washout. Consistently, the step 1_ON_ response decreased in cells expressing Gα_q_(Q209L) because of the independent binding of Gβγ to the receptor (Fig. 7c). However, the kinetics of step 1_ON_ and 1_OFF_ remained unchanged (Fig. 7e, g). These results further supported that the slow step 2_OFF_ in the FRETr response of hM3R-YFP plus Gα_q_-CFP was due to the slow dissociation of Gα_q_-CFP from hM3R-YFP. Through confocal microscopy imaging, we further confirmed that the cells transfected with hM3R-YFP, Gα_q_-CFP, and cognate G protein subunits also possessed a normal signaling cascade to PLCβ activation and PIP_2_ hydrolysis by receptor activation (Supplementary Fig. 19a). In addition, the hM3R signaling to PLCβ activation was sustained until washout of the agonist (Supplementary Fig. 19b).

To investigate the interaction of activated Gα_q_-GTP with its effector PLCβ, we performed FRETr experiments in cells transfected with hM3R, Gα_q_-CFP, YFP-PLCβ_1_, and cognate G protein subunits. The receptor activation upon Oxo-M application showed a single-step FRETr increase that was stronger but slower than that of hM3R-YFP and Gα_q_-CFP, suggesting that the coupling between hM3R and Gα_q_ is completed faster than the complex formation of Gα_q_ and PLCβ_1_ (Fig. 7h, i). Surprisingly, the increased FRETr signal between Gα_q_-CFP and YFP-PLCβ_1_ was also maintained during the activation of the receptor (Fig. 7h). When checking the deactivation process in Gα_q_-CFP plus YFP-PLCβ_1_, a single-step decrease was measured (Fig. 7h, j). The kinetics of this single-step deactivation was found to be slower than the step 1_OFF_ reaction of hM3R-YFP plus Gα_q_-CFP (Fig. 7g left) or hM3R-YFP-CFP (Supplementary Fig. 8a, d) but faster than the step 2_OFF_ reaction of hM3R-YFP plus Gα_q_-CFP (Fig. 7g, right) or hM3R-YFP-CFP (Supplementary Fig. 8a, e).

**Fig. 8:**
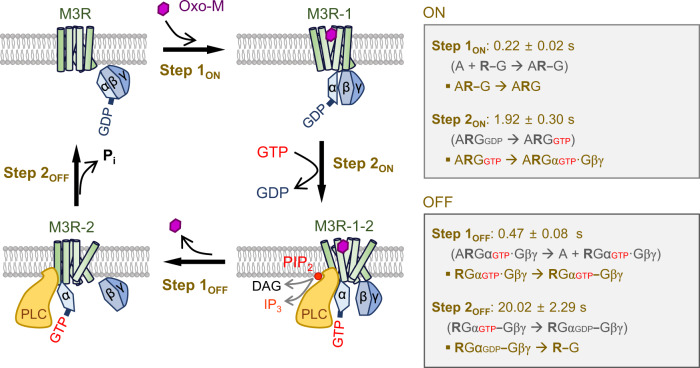
Schematic diagram of G_q_ protein cycle-mediated conformational changes of hM3R. Cartoon depicts the effects of G_q_ protein cycle on two-step activation and two-step deactivation of hM3R conformation. Boxes summarized the underlying processes of each step occurring during ON and OFF reactions. The reactions in the parentheses were not observed. The dash line (-) means a status of partial interaction or preassembly and a structural rearrangement by agonist binding induces more tighter binding. The binding of inactive G_q_ protein with the agonist-bound hM3R evokes a slight change in the coupling conformation between Gα_q_ and Gβγ subunits. The activated Gα_q_-GTP subunit does not dissociate from the ligand-activated hM3R, and it simultaneously stimulates PLCβ by forming a heteromeric complex.

Considering the stable interaction between active hM3R and Gα_q_-GTP (Fig. 7a), the results suggested that hM3R, Gα_q_, and PLCβ_1_ formed a stable heterotrimeric complex during receptor activation. To test this possibility, we examined the direct interaction between hM3R-CFP and YFP-PLCβ_1_. As shown in Fig. 7k, Oxo-M strongly increased the FRETr signal in cells expressing the two probes, supporting the conclusion that a stable complex formed between M3R and PLCβ_1_ upon receptor activation. The analysis of the FRETr signals revealed two-step responses during activation and deactivation (Fig. 7l, m, right). Interestingly, the kinetics of the fast steps 1_ON_ and 1_OFF_ was similar to that of receptor activation (Fig. 7l, m, left). The kinetics of step 2_OFF_ was close to that of the step 2_OFF_ of hM3R-YFP and Gα_q_-CFP (Fig. 7g, right), suggesting that PLCβ_1_ might dissociate from hM3R when Gα_q_ left the receptor. Together, the results demonstrate that PLCβ_1_ is released from Gα_q_-GTP more slowly than the step 1_OFF_ reaction and that this release ends before the completion of the step 2_OFF_ reaction (Fig. 8). Finally, to check the function of YM, we conducted the same FRETr experiments with Gα_q_-CFP plus YFP-PLCβ_1_ in the presence of YM (Supplementary Fig. 18b–f). There was no FRETr change by receptor activation, indicating that YM successfully inhibited G_q_ activation by blocking the GDP release from Gα_q_. The results further confirmed that the inactive G_q_ protein cannot interact with PLCβ_1_.

## Discussion

Various GPCR studies using FRET probes, bioluminescence resonance energy transfer (BRET) probes, or cpGFP (circular permutated green fluorescent protein) have been conducted to observe the intramolecular conformational change of receptors by ligand binding^36–38,46,55–61^. However, to observe the GPCR signaling pathways after receptor activation, additional experiments^36,38,62,63^ are required utilizing new photo probes corresponding to each pathway step. In those step-by-step experiments for the signaling cascades, the continuity of the entire structural deformation of GPCR is inevitably damaged. In this study, we overcame these limitations by constructing a M3R FRET sensor hM3R-YFP-CFP. Using the construct, we discovered that the structure of hM3R reversibly changed through a multistep process according to the activation cycle of the G_q_ protein. In addition, the FRET results among M3R, Gα_q_, and PLCβ suggested that the ligand-activated M3R formed a sustained heterotrimeric complex with Gα_q_ and PLCβ.

Here, we reported the two-step activation and deactivation of M3R. All existing fluorescence-based M3 sensors commonly displayed receptor activation through a single-step change in fluorescent signals. In previous studies, the activation of a receptor generally corresponds to the conversion of the M3R structure from an inactive state to an active one after agonist binding and not to the structural changes induced by G protein coupling. However, hM3R-YFP-CFP exhibited completely different properties from the existing sensors in that it detected G protein coupling after the activation stage and thus released the activation cycle of the G protein as a two-step FRET signal. This finding was further confirmed by the experiments involving the receptors with mutated Gα-binding sites without the impaired activation of the receptor^48^. The distinction in the target response of our receptor sensor might be attributed to the differences in receptor construction methods. For example, a fluoresceine arsenical hairpin binder (FlAsH)-based M3 FRET sensor^37,38^ shows normal downstream signaling similar to our hM3R-YFP-CFP but only detects the receptor activation through the FRET response, and the direction of response is increase that is opposite to ours. These differences might be due to the location and size of the inserted amino-acid motif CCPGCC, where the amino-acid residues from 271 and 465 (193 amino acids) of ICL3 were replaced with CCPGCCE (7 amino acids); in our construct, the residues between 270 and 485 (214 amino acids) were replaced with *Sac*II-EYFP-*Age*I (243 amino acids) in ICL3. In addition, the sensor required an additional binding of FlAsH to the inserted CCPGCC. Thus, such differences possibly led to the differential positioning of acceptor fluorescence (FlAsH or EYFP) relative to CFP between these two FRET sensors; eventually, the stage of signaling pathways and the direction of the FRET response varied. Similarly, GACh2.0 (ACh2.0)^46^ and GRAB_ACh3.0_ (ACh3.0)^45^ were constructed by inserting cpGFP into ICL3; when the receptor becomes activated, cpGFP transforms into an intact GFP structure and emits fluorescence. Unlike hM3R-YFP-CFP, this sensor is a dead form of downstream signaling and therefore can only detect receptor activation. This difference is also likely due to the locations of the inserted cpGFP instead of YFP. For ACh2.0, the residues between amino acids 253 and 493 (239 amino acids) of ICL3 were replaced with linker-cpGFP-linker (301 amino acids); for ACh3.0, the residues between amino acids 259 and 491 (231 amino acids) of ICL3 were replaced with linker-cpGFP-linker (246 amino acids). The ICL2 and ICL3 parts of GPCRs have conserved amino acids, which are essential for recognizing a certain type of G protein^24,64^. However, these amino acids were lost during cpGFP insertion; this loss likely caused these cpGFP-based sensors to become the dead form of downstream signaling.

Using hM3R-YFP-CFP, we demonstrated that a single-receptor FRET probe can reveal the multistep intramolecular conformational changes of a receptor during the coupled G protein cycle. We confirmed that, in the activation process, the association of ligand-activated hM3R with the inactive G_q_ protein displayed the fast step 1 FRETr signal and the following dissociation of Gα_q_ and Gβγ subunits under receptor coupling was responsible for the slower step 2 FRETr signal (Fig. 8). We could not obtain any independent FRETr change by ligand binding itself in hM3R-YFP-CFP even at a high frequency resolution (100 Hz) and fast solution exchange system (~20 ms). Previous studies have shown that ligand–M3R coupling is completed within 100 ms^37,62^, thus it is possible that the ligand binding to the external surface of our hM3R FRET construct generates relatively insignificant FRETr change.

In our disease model studies using the constitutively active Gα_q_(Q209L or R183C) forms, there was a single fast step of FRETr change in hM3R-YFP-CFP alone and in hM3R-CFP plus Gβ1-YFP. Since the Gβγ subunits exist independently in resting cells after decoupling from the constitutively active Gα_q_(Q209L or R183C), the results indicate that the ligand-activated hM3R can associate with the separated Gβγ subunits. In addition, the hM3R-Gβγ coupling remains unchanged during the YM application, confirming that the fast step 1 FRETr signal of hM3R-YFP-CFP in the presence of Gα_q_(Q209L or R183C) is mostly mediated by the intermolecular interaction with the Gβγ subunit. We also found that agonist binding to hM3R itself changes the interaction between Gα_q_ and Gβγ subunits before dissociation. As shown in Fig. 4i, j, even in the presence of the GDP release inhibitor YM, there was a minor but significant FRETr decrease between Gα_q_ and Gβγ subunits after Oxo-M application. These initial structural changes between Gα_q_ and Gβγ subunits upon binding to ligand-activated hM3R may be important for GDP release from the inactive Gα_q_ and subsequent GTP binding.

We confirmed that the loss of G protein coupling ability in the triple-alanine mutant (3x46/6x37/7x53) vasopressin V2 receptor^48^ can be reproduced in M3R, which is the same class A GPCR. By constructing hM3R(3A), we revealed that hM3R-YFP-CFP only detected the downstream signals related to the activation cycle of G_q_ protein but not the precedent receptor activation step by ligand binding. This was further confirmed by additional experiments using single-leucine mutant (3x50) M3R. Previous study reported that the mutant M3R(R165L^3x50^) still had normal agonist-binding affinity and arrestin-dependent signaling pathways^54^. Nevertheless, our hM3R(R166L^3x50^)-YFP-CFP showed no significant PIP_2_ hydrolysis and FRETr changes (Supplementary Fig. 17).

Another significant finding is the two-step deactivation of FRETr in cells expressing hM3R-YFP and Gα_q_-CFP after washout of the agonist. The fast step 1 and the subsequent slower step 2 recoveries seem to be due to the sequential interaction changes by the decoupling of the Gβγ subunit from hM3R following agonist dissociation and the dissociation of Gα_q_-CFP from hM3R after the slow dephosphorylation of GTP to GDP by GTPase activity, respectively. These results suggest that, in the recovery process from receptor activation, Gα_q_-GTP alone can be coupled with the receptor during the step 2 period even in the absence of the Gβγ subunit on the receptor.

Several studies related to the inactive-state preassembly of GPCRs and G proteins have been reported recently^24,26–33^. However, the results have been contradictory even when using the same M3R^33,65^. The discrepancy might be due to the fact that the studies could not directly probe the stability of the protein–protein complex^5^. In the present study, we showed that there was a significant FRETr increase between hM3R-CFP and Gβ1-YFP, indicating that the activated hM3R interacts with heterotrimeric G_q_ protein to form a tight complex. However, the FRETr signal amplitudes and the SNR were much smaller compared to the step 1 responses of hM3R-YFP-CFP. Those weak FRETr signals compared to noises can be caused by the preassembly of inactive hM3R with the Gβγ subunit of G_q_ protein in the resting state, although the association is not as tight as the condition under receptor activation. Thus, we speculate that agonist binding to hM3R may reorganize the preassembled complex to a closer conformation between the two probes.

It has generally been accepted that the activated Gα-GTP subunit dissociates from the receptor to yield a Gα-GTP monomer–effector complex, while the separated Gβγ subunit stays complexed with the receptor, which is now free to modulate other intracellular signaling molecules independently. However, some heterotrimeric G_i_ and G_s_ proteins maintain assembly with receptors in the early stage of activation^30,32^. Here, our study demonstrates that Gα_q_-GTP does not leave hM3R after dissociation from βγ subunits, and it continuously activates the downstream PLCβ molecule after forming a complex with hM3R until washout of the agonist. Interestingly, in our study, the increased FRETr signal between Gα_q_-CFP and YFP-PLCβ_1_ or between hM3R-CFP and YFP-PLCβ_1_ was also maintained steadily during the hM3R activation. These results are consistent with the sustained PIP_2_ depletion in the plasma membrane during the hM3R activation by the agonist (Supplementary Fig. 19). Therefore, in line with the previous studies showing that G protein can form a constitutive complex with effectors^66–70^, our results suggest that hM3R, Gα_q_, and PLCβ form a stable heterotrimeric complex together induced by the agonist and cause the steady PIP_2_ hydrolysis until washout of the agonist.

## Methods

### Constructs

In the plasmid (Addgene plasmid # 45547) encoding the human M3D receptor, two point mutations (C149Y and G239A) were created to generate wild-type human M3R (hM3R). This hM3R was appended to pcDNA 3, which includes cerulean, a variant of enhanced cyan fluorescent protein (ECFP), to generate hM3R-Cerulean. The restriction enzyme *Eco*RI recognition site mediated this appendage between Leu590 at the C terminus of hM3R and Met1 at the N terminus of cerulean. The cerulean of this hM3R-Cerulean was substituted with enhanced yellow fluorescent protein (EYFP) to generate hM3R-EYFP. To create the intramolecular fluorescent probe hM3R-EYFP-Cerulean, EYFP replaced a segment between Ala270 and Lys485 in the third intracellular loop of hM3R-Cerulean. The recognition sites of restriction enzymes *Sac*II and *Age*I mediated this replacement. Three amino-acid point mutations (I162A, L493A and Y544A) of these hM3R-Cerulean, hM3R-EYFP, and hM3R-EYFP-Cerulean were created to generate hM3R(3A)-Cerulean, hM3R(3A)-EYFP, and hM3R(3A)-EYFP-Cerulean. One amino-acid point mutation (R166L) of the hM3R-EYFP-Cerulean was created to generate hM3R(R166L^3x50^)-EYFP-Cerulean. Point mutations of the existing human Gα_q_^36^ and mouse Gα_q_-ECFP^36,71,72^ were created to generate Gα_q_(Q209L), Gα_q_(R183C), Gα_q_(Q209L)-ECFP, and Gα_q_(R183C)-ECFP. All constructs were made using adaptations of the QuikChange (Agilent Technologies, Santa Clara, CA) mutagenesis protocol and restriction enzyme kits (Enzynomics, Daejeon, South Korea) and were verified by automated sequencing (Macrogen, Seoul, South Korea). The sequences of primers used for plasmid cloning are listed in Supplementary Table 1. Mouse M1R-YFP-CFP^36^, mouse Gα_q_-ECFP^36,71,72^, bovine Gβ_1_-EYFP^36^, rat EYFP-PLCβ_1_^36,72^, and human pleckstrin homology (PH) domain probe RFP-PH(PLCδ_1_)^73^ were obtained from B. Hille (University of Washington School of Medicine, Seattle, WA). PH(PLCδ_1_)-ECFP and PH(PLCδ_1_)-EYFP^36,74^ were obtained from K. Jalink (The Netherlands Cancer Institute, Amsterdam, Netherlands). Plasmids containing unlabeled mouse M1R, human Gα_q_, Gβ_1_, Gγ_2_, and bovine GPCR kinase 2 (GRK2)^36^ were obtained from B. Hille. Here, we refer to fluorophores simply as CFP or YFP regardless of whether regular or enhanced fluorescent proteins were used. We confirm that all used unique biological materials are readily available from the corresponding author.

### Cell culture

Human embryonic kidney tsA-201 cells (large T-antigen transformed HEK293 cells (HEK293T cells); RRID: CVCL_2737) were a kind gift from Dr. Bertil Hille at University of Washington. The identity of this cell line has been authenticated by STR analysis and has recurrently tested negative for mycoplasma contamination using PCR (Cosmogenetech, Daejeon, South Korea). All experiments were performed on transiently transfected HEK293T cells. The 2-ml transfection medium contained 10 μl of Lipofectamine-2000 and 0.2–0.8 μg of each plasmid. In the G protein studies, for better membrane expression of any G protein subunit probe, cells were always transfected with three G protein subunits (α, β, and γ) together. The next day, the cells were plated onto poly-L-lysine-coated #0 glass coverslip chips, and fluorescent cells were studied 36–48 h after transfection.

### Förster resonance energy transfer (FRET)

Our FRET system was described in our previous publications^75–77^. FRET between CFP and YFP was measured by a photomultiplier tube (PMT)-based photometry system (Till Photonics GmbH). Regular pulses of indigo light (426–450 nm) from a homemade LED-based monochromator excited the fluorescent proteins. Emission, which passed through a 40×, NA 0.95 dry immersion objective lens (IX71; Olympus), was separated into short (460–500 nm) and long (520–550 nm) wavelengths by a dichroic mirror (505DCLP) and band-pass filters (D480-40 for short wavelengths and ET535-30 for long wavelengths; Chroma Technology) and detected by two PMTs connected with an FDU-2 fluorescence detection unit (Till Photonics GmbH). Donor and acceptor signals obtained by photometry system were transferred to a data acquisition board (PCI-6221; National Instruments, or EPC-10; HEKA Elektronik). Timing control, signal acquisition, and real-time calculation of FRET ratio (FRETr) including background compensation, were performed using a homemade program or PatchMaster (HEKA Elektronik) that also controlled the monochromator through the data acquisition interface. To correct bleed-through of emission of CFP into the YFP detector, cells expressing only CFP were used to obtain the ratio of the detected signal in short and long wavelength emission channels^36^. The calculated ratio (cFactor = CFP/YFP = 0.45) was used to correct the raw YFP emission signal. The bleed-through of YFP light into the CFP detector was only 0.02 and was neglected. The FRETr was thus calculated as follows:

$${{{{{\rm{FRETr}}}}}}=({{{{{\rm{YFP}}}}}}{{{{{\rm{c}}}}}}-{{{{{\rm{cFactor}}}}}}\times {{{{{\rm{CFP}}}}}}{{{{{\rm{c}}}}}})/{{{{{\rm{CFP}}}}}}{{{{{\rm{c}}}}}},$$

where YFP_C_ is the signal from YFP excited as a result of FRET (YFP emission by CFP excitation), CFP_C_ is the CFP emission detected by the short wavelength PMT, and YFP_C_ is the YFP emission detected by the long wavelength PMT.

The whole-cell configuration of the patch-clamp method using an EPC-10 patch-clamp amplifier at room temperature was used to manipulate intracellular concentration of GTP, GDPβS or GTPγS. Pipettes were pulled from glass micropipette capillaries (World Precision Instruments) using a Flaming/Brown micropipette puller (P-97; Sutter Instrument Co.). Pipette resistance was 1.3–3.0 MΩ, and series resistance was between 3.4 and 6.0 MΩ with 60% compensation. The pipette solution composition was described in Solutions and materials section. The holding potential in all experiments was −80 mV. The raw data were processed with Excel 2016 (Microsoft).

### Confocal imaging

HEK293T cells were imaged 2 d after transfection on poly-L-lysine-coated chips with a LSM 700 confocal microscope (Carl Zeiss) at room temperature. The images were scanned with a 40 × objective lens at 1024 × 1024 or 512 × 512 pixels using digital zoom on a cover glass-bottom dish filled with the external solution. Image processing and measurement of fluorescence intensity were carried out using Zeiss ZEN 2.3 SP1 software. To analyze relative fluorescence intensity, the fluorescence intensity of a region of interest (ROI) at each time point was divided by the average of points 30-s period before Oxo-M or Ach treatment. The half time of activation curve (*T*_50_) was calculated from sigmoidal curve fitting. All images were transferred from LSM4 to JPG format. The raw data were processed with Excel 2016 (Microsoft).

### Solutions and materials

Cells were subjected to a continuous slow bath flow of Ringer’s solution. The fresh external Ringer’s solution was supplied to the chamber by the valve-controlled gravity perfusion system VC3-4CLG from ALA Scientific Instruments (Farmingdale, New York). This supplied solution was simultaneously sucked out by the vacuum pump system DR 4-000-007 from Drummond Scientific Company (Broomall, Pennsylvania). The external Ringer’s solution exchange for drug treatment was accomplished by a theta tube moved laterally by a step-driven motor (SF-77C) from Warner Instruments (Holliston, Massachusetts) and was complete within 20 ms. The external Ringer’s solution used for FRET and confocal imaging contained the following (in mM): 160 NaCl, 2.5 KCl, 2 CaCl_2_, 1 MgCl_2_, 10 HEPES, and 8 glucose, adjusted to pH 7.4 with NaOH. The pipette solution for FRET under the normal GTP concentration (0.1 mM) condition contained the following (in mM): 175 KCl, 5 MgCl_2_, 5 HEPES, 0.1 BAPTA, 3 Na_2_ATP, and 0.1 Na_3_GTP, adjusted to pH 7.4 with KOH. For the case of the GTP depletion condition, the 0.1 mM Na_3_GTP was substituted with 1 mM GDPβS. For the case of the nonhydrolyzable GTP analog experiments, the 0.1 mM GTP was substituted with 0.1 mM GTPγS. When using Oxo-M as an agonist, a concentration of 10 μM was used. Oxo-M, Ach and poly-l-lysine were from Sigma-Aldrich (St. Louis, Missouri). DMEM, Lipofectamine-2000, and penicillin/streptomycin were from Invitrogen (Waltham, Massachusetts). Fetal bovine serum was from Gemini Bio-Products (West Sacramento, California). YM-254890 was from Cayman Chemical (Ann Arbor, Michigan).

### In silico modeling using I-TASSER and SWISS-Model

For the predicted three-dimensional (3D) structure of M3R with mutating Gα-binding sites, molecular modeling was executed by using the cryo-EM structure of rat M3R (PDB ID: 4DAJ)^53^ as a template from Protein Data Bank (PDB) because there is no cryo-EM structure of human M3R. In Swiss-Model, we used the structure with PDB ID: 4U14 determined by X-ray crystallography as template^78^. We submitted the full sequence of rat M3R(3A) (derived from NM_012527.2) and obtained models predicted from I-TASSER online server V5.2 (http://zhanglab.ccmb.med.umich.edu/I-TASSER/)^79^ and SWISS-Model web server released from July 2022 (https://swissmodel.expasy.org/). Excluding the flexible intracellular loops, transmembrane domains with Gα-binding sites of cryo-EM M3R and in silico M3R(3A) model were superimposed by matchmaker of University of California, San Francisco (UCSF) chimera. The amino-acid residues representing orthosteric agonist-binding sites^53,80^ and Gα-binding sites^48,52^ were indicated. Structure visualization and modifications were made using UCSF chimera.

### Data analysis

All data were analyzed using Excel 2016 (Microsoft), Igor Pro-6.0 (WaveMetrics), or GraphPad Prism 8 (GraphPad Software). The measured FRETr traces were normalized as follows. The average FRETr signal of 5-s period before the agonist treatment was set to the initial value 1. The average FRETr signal of 5-s period before the washing out of drugs was set to the minimum 0 or maximum 2 value according to the direction of the reactions. To measure the relative FRETr in a time course, the data at each time point were divided by the average FRETr signal of 5-s period before the agonist treatment. To perform a fitting and determine a time constant (τ) and ΔFRETr amplitude of activation in the “hM3R-YFP-CFP” experiments, when the starting point of agonist treatment was regarded as the zero time point, the time duration from 0 to 1.2 s after agonist addition was designated as “Step 1_ON_” and the 1.3–15 s time period during the agonist treatment was designated as “Step 2_ON_”. To perform a fitting and determine a time constant (τ) of deactivation in the “hM3R-YFP + Gα_q_-CFP” experiments, 0 − 3.6 s was designated as “Step 1_OFF_” and 3.7–30 s was designated as “Step 2_OFF_”. Except for the cases previously described, a fitting was performed with a least-squares criterion to determine time constants (τ) of activation and deactivation and the maximum ΔFRETr amplitude. The signal-to-noise ratio (SNR) was calculated as the maximum ΔFRETr amplitude divided by the standard error of the baseline ΔFRETr. Percent (%) ON of FRETr of hM3R-YFP-CFP was calculated as 100× (ΔFRETr amplitude of each activation step (step 1 or step 2)/maximum ΔFRETr amplitude). Percent (%) ON of FRETr of the other constructs with single-step activation was calculated as 100× (the average of the amplitudes (in contrast to the initial value 1) of points of 2.5-s period before washing out of agonist in each normalized FRETr/the average of the amplitudes (in contrast to the initial value 1) of points of 2.5-s period before agonist washing out in normalized average FRETr). Percent (%) OFF of FRETr of hM3R-YFP-CFP was calculated as 100× (ΔFRETr amplitude of each deactivation step (step 1 or step 2)/maximum ΔFRETr amplitude of activation). Percent (%) OFF of FRETr of the other constructs with single-step deactivation was calculated as 100× (ΔFRETr amplitude of deactivation/maximum ΔFRETr amplitude of activation). Statistics in the text and figures represent mean ± SEM. Statistical comparisons between the means of two groups were analyzed using Student’s *t*-test and Welch’s *t*-test according to their variances. Otherwise, Mann–Whitney *U* test was used if the data did not follow normal distribution. Statistical significances of ΔFRETr changes before and after receptor activations were analyzed using paired *t*-test. Statistical comparisons between the means of three or more independent groups under one independent variable were analyzed using one-way ANOVA or Welch’s ANOVA according to their variances. Statistical comparisons between the means of three or more independent groups with equal variance under two independent variables were analyzed using two-way ANOVA. Differences were considered significant at the *P < 0.05, **P < 0.01, ***P < 0.001, and ****P < 0.0001 levels. Source data used in data analysis are provided with this paper.

### Reporting summary

Further information on research design is available in the Nature Portfolio Reporting Summary linked to this article.

## Supplementary information

Supplementary Information

Reporting Summary

## Data Availability

The data that support this study are available from the corresponding authors upon request. The published cryo-EM structure of rat M3R can be accessed using accession code 4DAJ. The published X-ray crystal structure of rat M3R can be accessed using accession code 4U14. Source data are provided with this paper.

## Source data

Source Data
