# Supplementary material for: Two-step structural changes in M3 muscarinic receptor activation rely on the coupled Gq protein cycle
Source: Nat Commun. 2023 Mar 8;14:1276. doi: 10.1038/s41467-023-36911-4 (PMC9992711; doi:10.1038/s41467-023-36911-4)
Supplement: Supplementary file 1 — Supplementary Information [file 41467_2023_36911_MOESM1_ESM.pdf]

Supplementary Information

Two-step structural changes in M3 muscarinic receptor activation rely on the coupled G_q protein cycle

Yong-Seok Kim, Jun-Hee Yeon, Woori Ko & Byung-Chang Suh*

19 Supplementary Figures
1 Supplementary Table

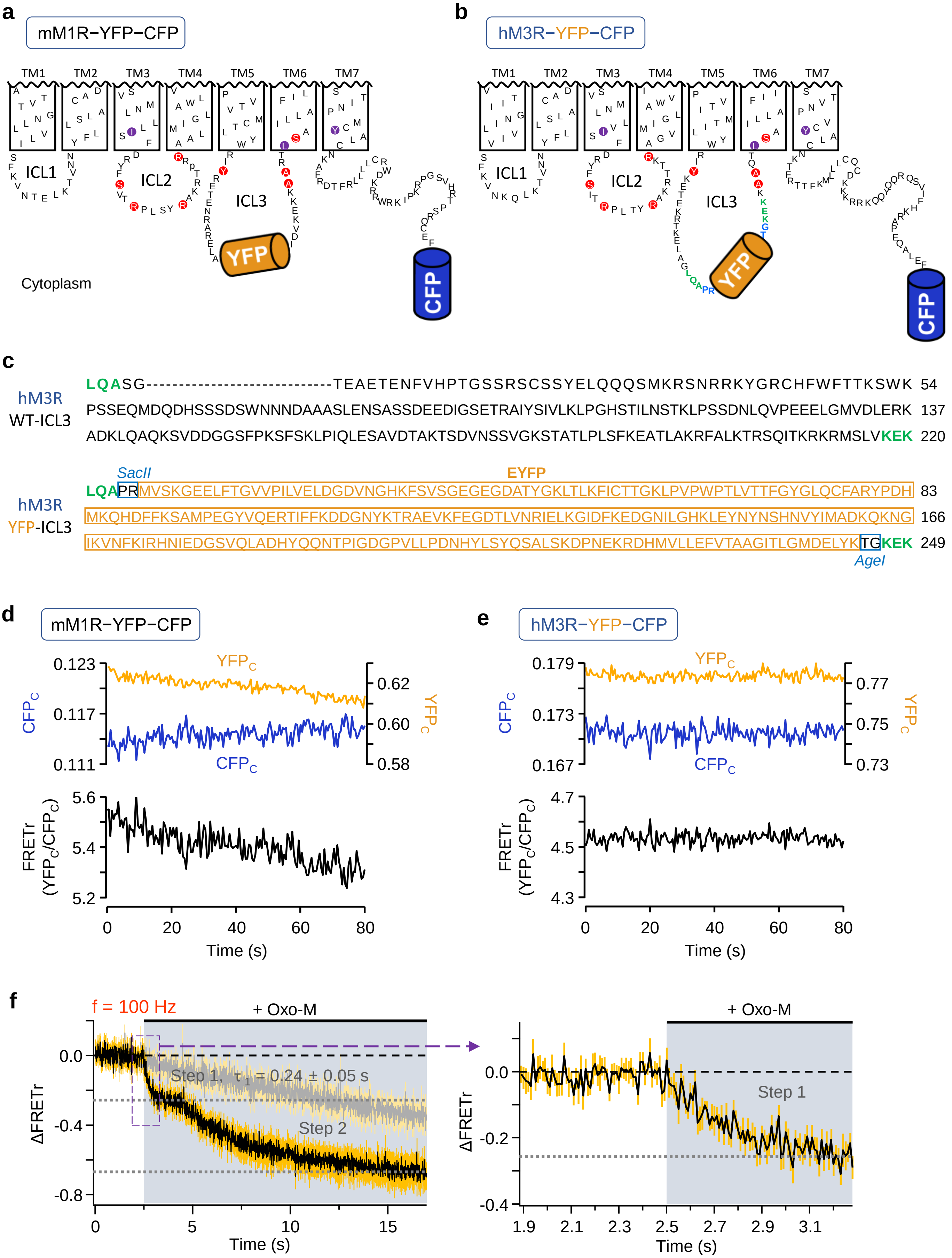
a
mM1R−YFP−CFP
TM1
TM2
TM3
TM4
TM5
TM6
TM7
ICL1
ICL2
ICL3
YFP
CFP
Cytoplasm
b
hM3R−YFP−CFP
TM1
TM2
TM3
TM4
TM5
TM6
TM7
ICL1
ICL2
ICL3
YFP
CFP
c
hM3R
WT-ICL3
LQASG----------------------------TEAETENFVHPTGSSRSCSSYELQQQSMKRSNRRKYGRCHFWFTTKSWK 54
PSSEQMDQDHSSSDSWNNNDAASLENSASSDEEDIGSETRAIYSIVLKLPGHSTILNSTKLPSSDNLQVPEEELGMVDLERK 137
ADKLQAQKSVDDGGSFPKSFSKLPIQLESAVDTAKTSDVNSSVGKSTATLPLSFKEATLAKRFALKTRSQITKRKRMSLVKEK 220
SacII
EYFP
hM3R
YFP-ICL3
LQAPRMVSKGEELFTGVVPILVELDGDVNGHKFSVSGEGEGDATYGKLTLKFICTTGKLPVPWPTLVTTFGYGLQCFARYPDH 83
MKQHDFFKSAMPEGYVQERTIFFKDDGNYKTRAEVKFEGDTLVNRIELKGIDFKEDGNILGHKLEYNYNSHNVYIMADKQKNG 166
IKVNFKIRHNIEDGSVQLADHYQQNTPIGDGPVLLPDNHYLSYQSALSKDPNEKRDHMVLLEFVTAAGITLGMDELYKTGKEK 249
AgeI
d
mM1R−YFP−CFP
YFPC
CFPC
0.123
0.117
0.111
0.62
0.60
0.58
FRETr (YFPC/CFPC)
5.6
5.4
5.2
0
20
40
60
80
Time (s)
e
hM3R−YFP−CFP
YFPC
CFPC
0.179
0.173
0.167
0.77
0.75
0.73
FRETr (YFPC/CFPC)
4.7
4.5
4.3
0
20
40
60
80
Time (s)
f
f = 100 Hz
+ Oxo-M
Step 1, τ1 = 0.24 ± 0.05 s
Step 2
ΔFRETr
0.0
-0.4
-0.8
0
5
10
15
Time (s)
+ Oxo-M
Step 1
ΔFRETr
0.0
-0.2
-0.4
1.9
2.1
2.3
2.5
2.7
2.9
3.1
Time (s)

Supplementary Figure 1. Construction of hM3R-YFP-CFP and FRET signal. Schematic transmembrane topology of mM1R-YFP-CFP (**a**) and hM3R-YFP-CFP (**b**). The amino acids highlighted in red are predicted to be selective recognition sites of each subtype for $G_{q/11}$-protein[64]. The amino acids highlighted in purple are residues in class A GPCRs which are rearranged by receptor activation to release Gα subunit-contacting residues[48]. **c**, Comparison of the third intracellular loop (ICL3) amino acid sequences between wild type hM3R ICL3 and hM3R-YFP-CFP ICL3 (YFP-ICL3). 214 amino acid residues in ICL3 were replaced with 243 residues of *Sac*II-EYFP-*Age*I sequence. The amino acids highlighted in green correspond to the green-highlighted sequences in (**b**). The highlighted in blue squares in YFP-ICL3 are restriction enzyme sites for inserting the EYFP. The EYFP sequence is highlighted in a yellow square box. Time-course traces of CFPc, YFPc and FRETr signals in cells transfected with mM1R-YFP-CFP (**d**) or hM3R-YFP-CFP (**e**). The top panel shows corrected CFP (CFP_C) fluorescence (blue trace, left axis) and corrected YFP (YFP_C) fluorescence (yellow trace, right axis), and the bottom panel shows the FRET ratio, YFC_C/CFP_C (black), for 80-s baseline. Sampling frequency: 2 Hz. **f,** Time course of ΔFRETr measured in high frequency (100 Hz) in intact cells expressing hM3R-YFP-CFP. Yellow vertical lines indicate SEM. Oxo-M (10 μM) was treated to the cells by using the fast solution exchange system (< 20 ms). The FRETr changes by Oxo-M application show two-step activation decays (step 1 and 2). Step1 was fitted by a single-exponential function to calculate a time constant (τ_1). n = 12 cells from two cultures. Blur run-down graph indicates data from cells not treated with Oxo-M (n = 11 cells from two cultures). Zoomed in view of boxed region (step 1) in left graph is shown on the right. Source data are provided as a Source Data file.

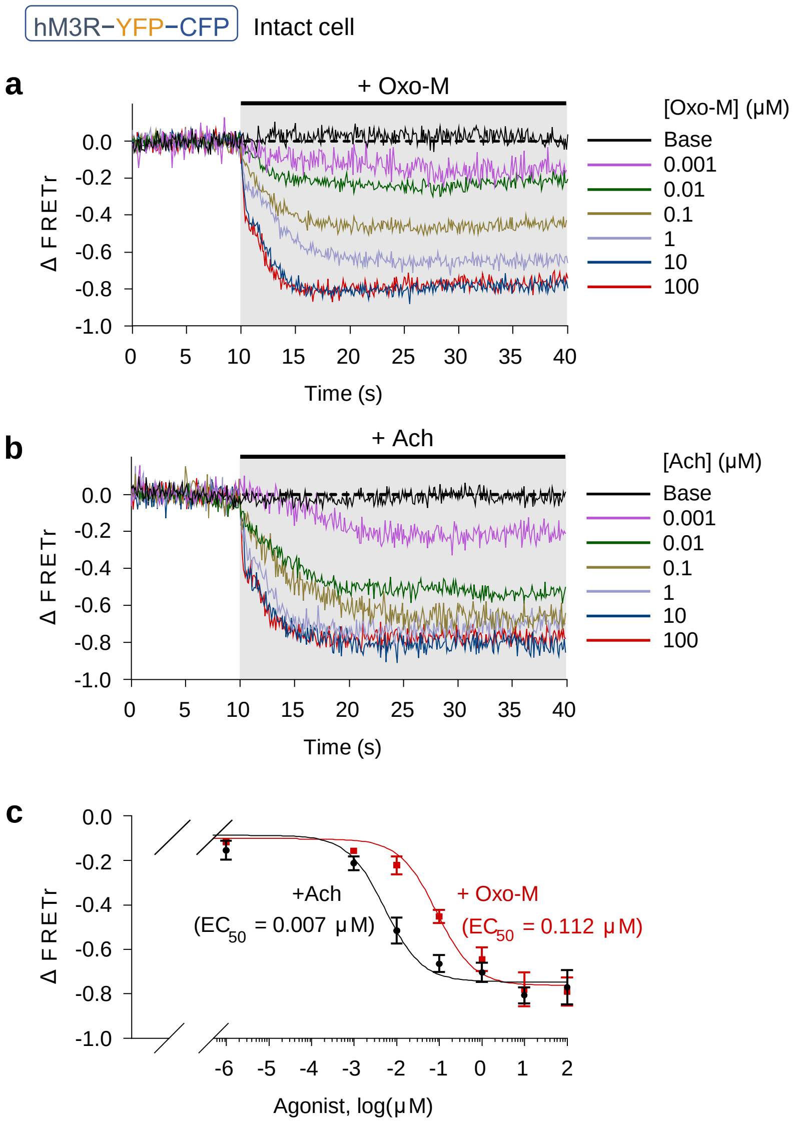

Supplementary Figure 2. Relationship of agonist concentration to ΔFRETr response of hM3R-YFP-CFP. a, Time course of mean ΔFRETr in response to each concentration of Oxo-M in cells expressing hM3R-YFP-CFP. Sampling frequency: 10 Hz. n = 10 cells from 2 cultures for Base; n = 5 cells from two cultures for 0.001 μM Oxo-M; n = 8 cells from two cultures for 0.01 μM Oxo-M; n = 10 cells from two cultures for 0.1 μM Oxo-M; n = 9 cells from two cultures for 1 μM Oxo-M; n = 8 cells from three cultures for 10 μM Oxo-M; n = 6 cells from three cultures for 100 μM Oxo-M. **b**, Time courses of mean ΔFRETr in response to each concentration of acetylcholine (Ach) in cells expressing hM3R-YFP-CFP. Sampling frequency: 10 Hz. n = 10 cells from two cultures for Base; n = 13 cells from two cultures for 0.001 μM Ach; n = 15 cells from two cultures for 0.01 μM Ach; n = 5 cells from two cultures for 0.1 μM Ach; n = 6 cells from two cultures for 1 μM Ach; n = 5 cells from two cultures for 10 μM Ach; n = 6 cells from two cultures for 100 μM Ach. **c**, Dose-dependent ΔFRETr change of hM3R-YFP-CFP by Oxo-M and Ach. n = 5 cells from two cultures for 1 pM Oxo-M. n = 7 cells from two cultures for 1 pM Ach. Data are shown as mean ± SEM. Source data are provided as a Source Data file.

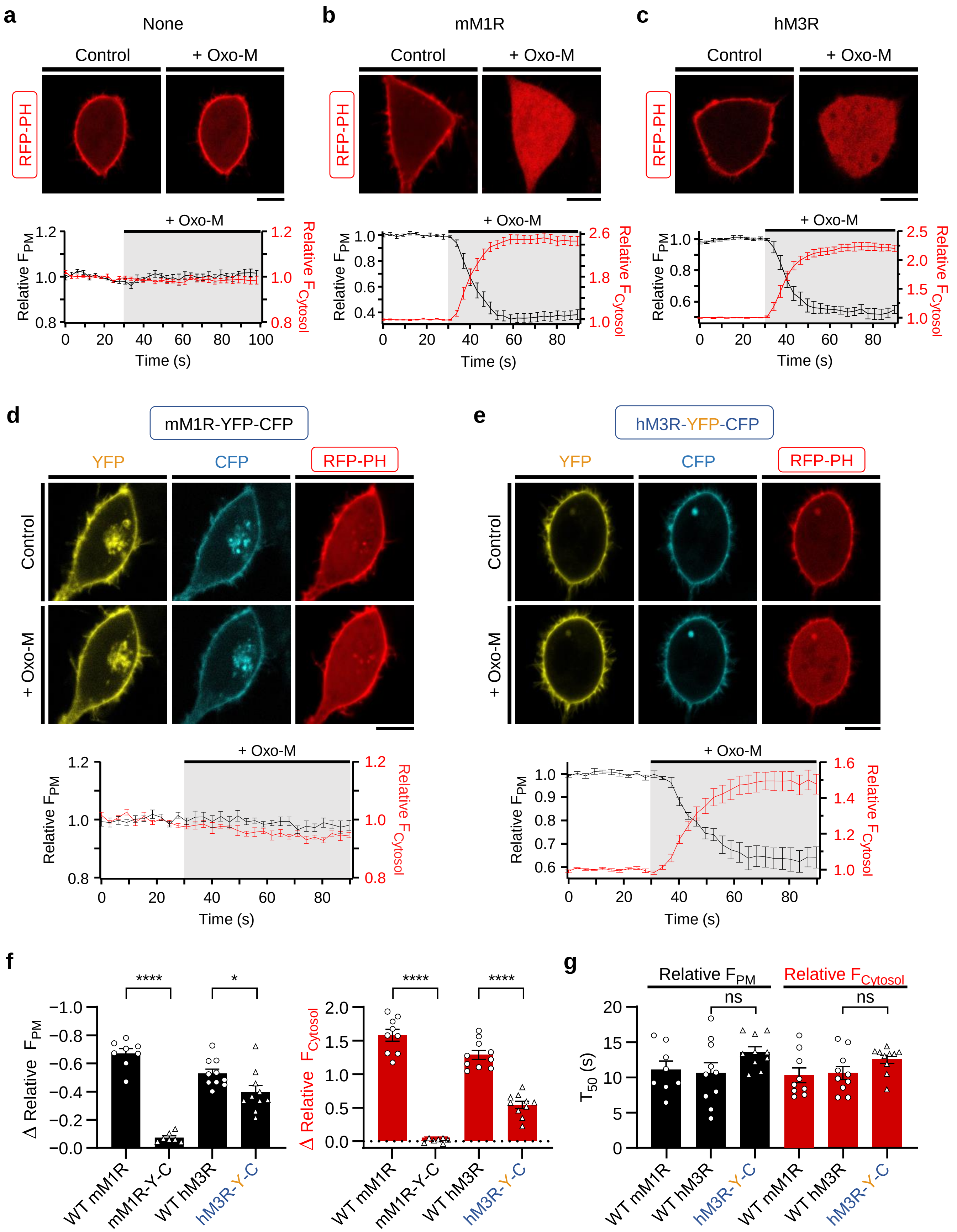

a
None
Control
+ Oxo-M
RFP-PH
b
mM1R
c
hM3R
Relative F_{PM}
Relative $F_{Cytosol}$
Time (s)
d
mM1R-YFP-CFP
YFP
CFP
RFP-PH
e
hM3R-YFP-CFP
f
****
*
Δ Relative F_{PM}
Δ Relative $F_{Cytosol}$
WT mM1R
mM1R-Y-C
WT hM3R
hM3R-Y-C
g
ns
T_{50} (s)

Supplementary Figure 3. Activation of hM3R-YFP-CFP triggers downstream signaling pathways with similar kinetics to the wild type hM3R. a-c, Top, representative confocal images of cells expressing red fluorescent protein-labelled pleckstrin homology domain of PLCδ1 (RFP-PH) without (none) or with wild type receptor (mM1R or hM3R) were taken before (Control) and during 10 μM Oxo-M application. Scale bar, 10 μm. Bottom, time courses of relative fluorescence intensity of RFP-PH at plasma membrane (F_{PM}, black trace, left axis) and cytosol ($F_{Cytosol}$, red trace, right axis) were measured in 7 (none), 9 (mM1R), and 10 (hM3R) cells from three (none and mM1R) and four (hM3R) independent experiments. Sampling frequency: 0.33 Hz. Top, representative confocal images of cells expressing RFP-PH and mM1R-YFP-CFP (**d**) or hM3R-YFP-CFP (**e**) were obtained before (Control) and during Oxo-M application. Scale bar, 5 μm. Bottom, time courses of relative fluorescence intensity of RFP-PH at plasma membrane (black trace, left axis) and cytosol (red trace, right axis) were measured in 7 (mM1R-YFP-CFP) and 10 (hM3R-YFP-CFP) cells from three (mM1R-YFP-CFP) and four (hM3R-YFP-CFP) independent experiments. Sampling frequency: 0.33 Hz. **f**, Variance (Δ) of relative fluorescence intensity of RFP-PH at plasma membrane (F_{PM}) and cytosol ($F_{Cytosol}$) before and after Oxo-M application under each receptor condition. WT mM1R, n = 9 cells from three independent experiments; mM1R-YFP-CFP, n = 7 cells from three independent experiments; WT hM3R, n = 10 cells from three independent experiments; hM3R-YFP-CFP, n = 10 cells from four independent experiments. In plasma membrane data, statistical significances were determined using Welch's t test (WT mM1R vs mM1R-YFP-CFP) (two-sided, $p < 0.0001$), and Student's t test (WT hM3R vs hM3R-YFP-CFP) (two-sided, $p = 0.0291$). In cytosol data, statistical significances were determined using Welch's t test (WT mM1R vs mM1R-YFP-CFP) (two-sided, $p < 0.0001$), and Student's t test (WT hM3R vs hM3R-YFP-CFP) (two-sided, $p < 0.0001$). **g**, The half activation time (T_{50}) of the relative fluorescence intensity curves of RFP-PH at plasma membrane and cytosol during Oxo-M application under each receptor condition. WT mM1R, n = 9 cells from three independent experiments; WT hM3R, n = 10 cells from three independent experiments; hM3R-YFP-CFP, n = 10 cells from four independent experiments. In plasma membrane data, statistical significance was determined using Welch's t test Student's t test (WT hM3R vs hM3R-YFP-CFP) (two-sided). In cytosol data, statistical significance was determined using Student's t test (WT hM3R vs hM3R-YFP-CFP) (two-sided). Data are shown as mean ± SEM. *$P<0.05$; ****$P<0.0001$; ns, not significant. Source data are provided as a Source Data file.

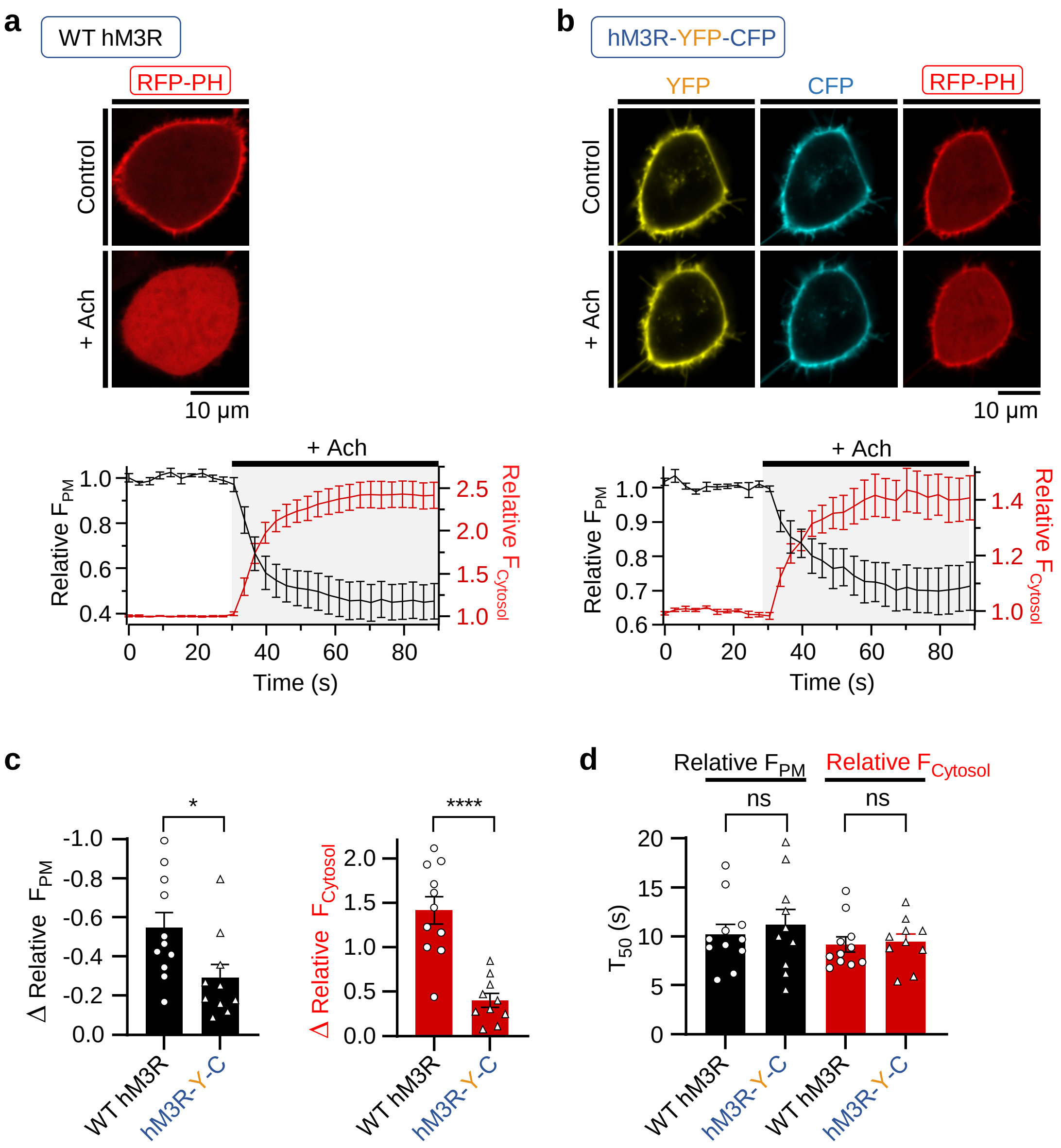

Supplementary Figure 4. Activation of hM3R-YFP-CFP by acetylcholine triggers downstream signaling pathways with similar kinetics to the wild type hM3R. Top, representative confocal images of cells expressing RFP-PH and wild-type hM3R (**a**) or hM3R-YFP-CFP (**b**) were obtained before (Control) and during 10 µM Ach application. Scale bar, 10 µm. Bottom, time courses of relative fluorescence intensity of RFP-PH at plasma membrane (black trace, left axis) and cytosol (red trace, right axis) were measured in 11 (hM3R) and 10 (hM3R-YFP-CFP) cells from two independent experiments. Sampling frequency: 0.33 Hz. **c**, Variance (Δ) of relative fluorescence intensity of RFP-PH at plasma membrane (F_{PM}) and cytosol ($F_{Cytosol}$) before and after Ach application under each receptor condition. WT hM3R, n = 11 cells from two independent experiments; hM3R-YFP-CFP, n = 10 cells from two independent experiments. In plasma membrane data, statistical significance was determined using Student's t test (two-sided, $p = 0.0269$). In cytosol data, statistical significance was determined using Welch's t test (two-sided, $p < 0.0001$). **d**, The half activation time (T_{50}) of the relative fluorescence intensity curves of RFP-PH at plasma membrane and cytosol during Ach application under each receptor condition. WT hM3R, n = 11 cells from two independent experiments; hM3R-YFP-CFP, n = 10 cells from two independent experiments. Statistical significances were determined using Student's t test (two-sided). Data are shown as mean ± SEM. *P<0.05; ****P<0.0001; ns, not significant. Source data are provided as a Source Data file.

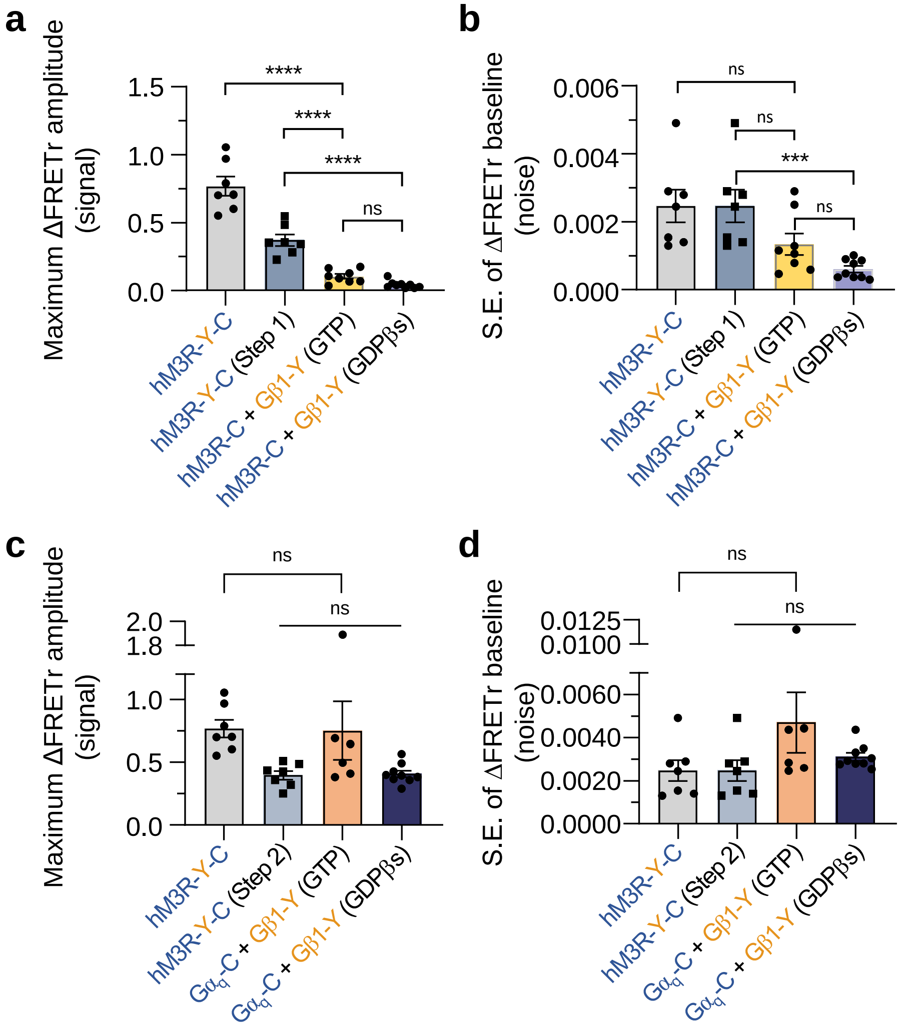
a
b
c
d
Maximum ΔFRETr amplitude (signal)
S.E. of ΔFRETr baseline (noise)
****
ns
***
hM3R-Y-C
hM3R-Y-C (Step 1)
hM3R-C + Gβ1-Y (GTP)
hM3R-C + Gβ1-Y (GDPβs)
hM3R-Y-C (Step 2)
Gα$_q$-C + Gβ1-Y (GTP)
Gα$_q$-C + Gβ1-Y (GDPβs)

Supplementary Figure 5. Peak response (signal) and standard error of baseline (noise). **a**, Peak response of ΔFRETr in response to Oxo-M application in cells expressing each FRET sensor. Except for the cells belonging to the fourth column intracellularly perfused with 1 mM GDPβS, all cells belonging to the other columns were intracellularly perfused with 0.1 mM GTP. hM3R-YFP-CFP (step 1 + step 2) and hM3R-YFP-CFP (step 1), n = 7 cells from three cultures; hM3R-CFP + Gβ1-YFP (GTP), n = 8 cells from four cultures; hM3R-CFP + Gβ1-YFP (GDPβS), n = 9 cells from three cultures. Statistical significances were determined using Welch's t test (hM3R-YFP-CFP (step 1 + step 2) vs hM3R-CFP + Gβ1-YFP (GTP)) (two-sided, p < 0.0001), and one-way ANOVA test with Tukey post hoc test (p < 0.0001; hM3R-YFP-CFP (step 1) vs hM3R-CFP + Gβ1-YFP (GTP) p < 0.0001, hM3R-YFP-CFP (step 1) vs hM3R-CFP + Gβ1-YFP (GDPβS) p < 0.0001, hM3R-CFP + Gβ1-YFP (GTP) vs hM3R-CFP + Gβ1-YFP (GDPβS) p = 0.1712). **b**, Standard error of ΔFRETr baseline in cell expressing each FRET sensor. hM3R-YFP-CFP (step 1 + step 2) and hM3R-YFP-CFP (step 1), n = 7 cells from three cultures; hM3R-CFP + Gβ1-YFP (GTP), n = 8 cells from four cultures; hM3R-CFP + Gβ1-YFP (GDPβS), n = 9 cells from three cultures. Statistical significances were determined using Student's t test (hM3R-YFP-CFP (step 1 + step 2) vs hM3R-CFP + Gβ1-YFP (GTP)) (two-sided, p = 0.0648), and one-way ANOVA test with Tukey post hoc test (p = 0.0014; hM3R-YFP-CFP (step 1) vs hM3R-CFP + Gβ1-YFP (GTP) p = 0.0509, hM3R-YFP-CFP (step 1) vs hM3R-CFP + Gβ1-YFP (GDPβS) p = 0.0010, hM3R-CFP + Gβ1-YFP (GTP) vs hM3R-CFP + Gβ1-YFP (GDPβS) p = 0.2095). **c**, Peak response of ΔFRETr in response to Oxo-M application in cells expressing each FRET sensor. Except for the cells belonging to the fourth column intracellularly perfused with 1 mM GDPβS, all cells belonging to the other columns were intracellularly perfused with 0.1 mM GTP. hM3R-YFP-CFP (step 1 + step 2) and hM3R-YFP-CFP (step 2 only), n = 7 cells from three cultures; $G\alpha_q$-CFP + Gβ1-YFP (GTP), n = 6 cells from two cultures; $G\alpha_q$-CFP + Gβ1-YFP (GDPβS), n = 9 cells from three cultures. Statistical significances were determined using Welch's t test (hM3R-YFP-CFP (step 1 + step 2) vs Gαq-CFP + Gβ1-YFP (GTP)) (two-sided), and one-way ANOVA test with Tukey post hoc test (p = 0.0787; hM3R-YFP-CFP (step 2) vs Gαq-CFP + Gβ1-YFP (GTP) p = 0.1137, hM3R-YFP-CFP (step 2) vs Gαq-CFP + Gβ1-YFP (GDPβS) p = 0.9971, Gαq-CFP + Gβ1-YFP (GTP) vs Gαq-CFP + Gβ1-YFP (GDPβS) p = 0.1040. **d**, Standard error of ΔFRETr baseline in cell expressing each FRET sensor. hM3R-YFP-CFP (step 1 + step 2) and hM3R-YFP-CFP (step 2 only), n = 7 cells from three cultures; $G\alpha_q$-CFP + Gβ1-YFP (GTP), n = 6 cells from two cultures; $G\alpha_q$-CFP + Gβ1-YFP (GDPβS), n = 9 cells from three cultures. Statistical significances were determined using Welch's t test (hM3R-YFP-CFP (step 1 + step 2) vs Gαq-CFP + Gβ1-YFP (GTP)) (two-sided), and one-way ANOVA test with Tukey post hoc test (p = 0.1349; hM3R-YFP-CFP (step 2) vs Gαq-CFP + Gβ1-YFP (GTP) p = 0.1250, hM3R-YFP-CFP (step 2) vs Gαq-CFP + Gβ1-YFP (GDPβS) p = 0.7923, Gαq-CFP + Gβ1-YFP (GTP) vs Gαq-CFP + Gβ1-YFP (GDPβS) p = 0.2909. Data are shown as mean ± SEM. ***P<0.001; ****P<0.0001; ns, not significant. Source data are provided as a Source Data file.

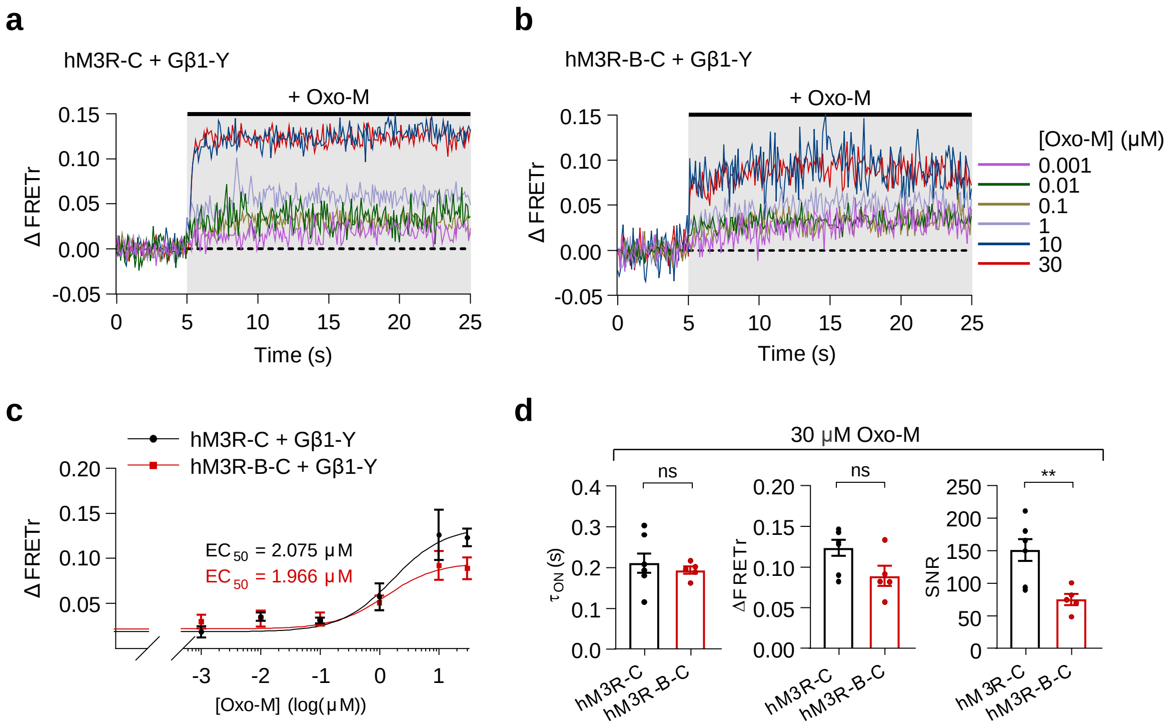

Supplementary Figure 6. Relationship of agonist concentration to ΔFRETr response of hM3R and Gβγ subunits. a, Time course of mean ΔFRETr in response to each concentration of Oxo-M in cells expressing hM3R-CFP, Gβ1-YFP, Gα_q and Gγ2. Sampling frequency: 10 Hz. **b**, Time course of mean ΔFRETr in response to each concentration of Oxo-M in cells expressing hM3R-BFP-CFP, Gα_q, Gβ1-YFP, and Gγ2. Sampling frequency: 10 Hz. **c**, Dose-dependent ΔFRETr changes in cells expressing hM3R-CFP and Gβ1-YFP (n = 11 cells from two cultures for 0.001 μM; n = 6 cells from two cultures for 0.01 μM; n = 10 cells from two cultures for 0.1 μM; n = 10 cells from two cultures for 1 μM; n = 5 cells from two cultures for 10 μM; n = 7 cells from two cultures for 30 μM) or hM3R-BFP-CFP and Gβ1-YFP (n = 13 cells from two cultures for 0.001 μM; n = 7 cells from two cultures for 0.01 μM; n = 10 cells from two cultures for 0.1 μM; n = 10 cells from two cultures for 1 μM; n = 8 cells from two cultures for 10 μM; n = 5 cells from two cultures for 30 μM). **d**, Time constant (τ_{ON}), ΔFRETr, and SNR of the FRETr responses induced by 30 μM Oxo-M in cells expressing each FRET sensor. hM3R-CFP, n = 7 cells from two cultures; hM3R-BFP-CFP, n = 5 cells from two cultures. In τ_{ON} data, statistical significance was determined using Welch's t test (two-sided). In ΔFRETr data, statistical significance was determined using Student's t test (two-sided). In SNR data, statistical significance was determined using Student's t test (two-sided, p = 0.0053). Data are shown as mean ± SEM. **P<0.01; ns, not significant. Source data are provided as a Source Data file.

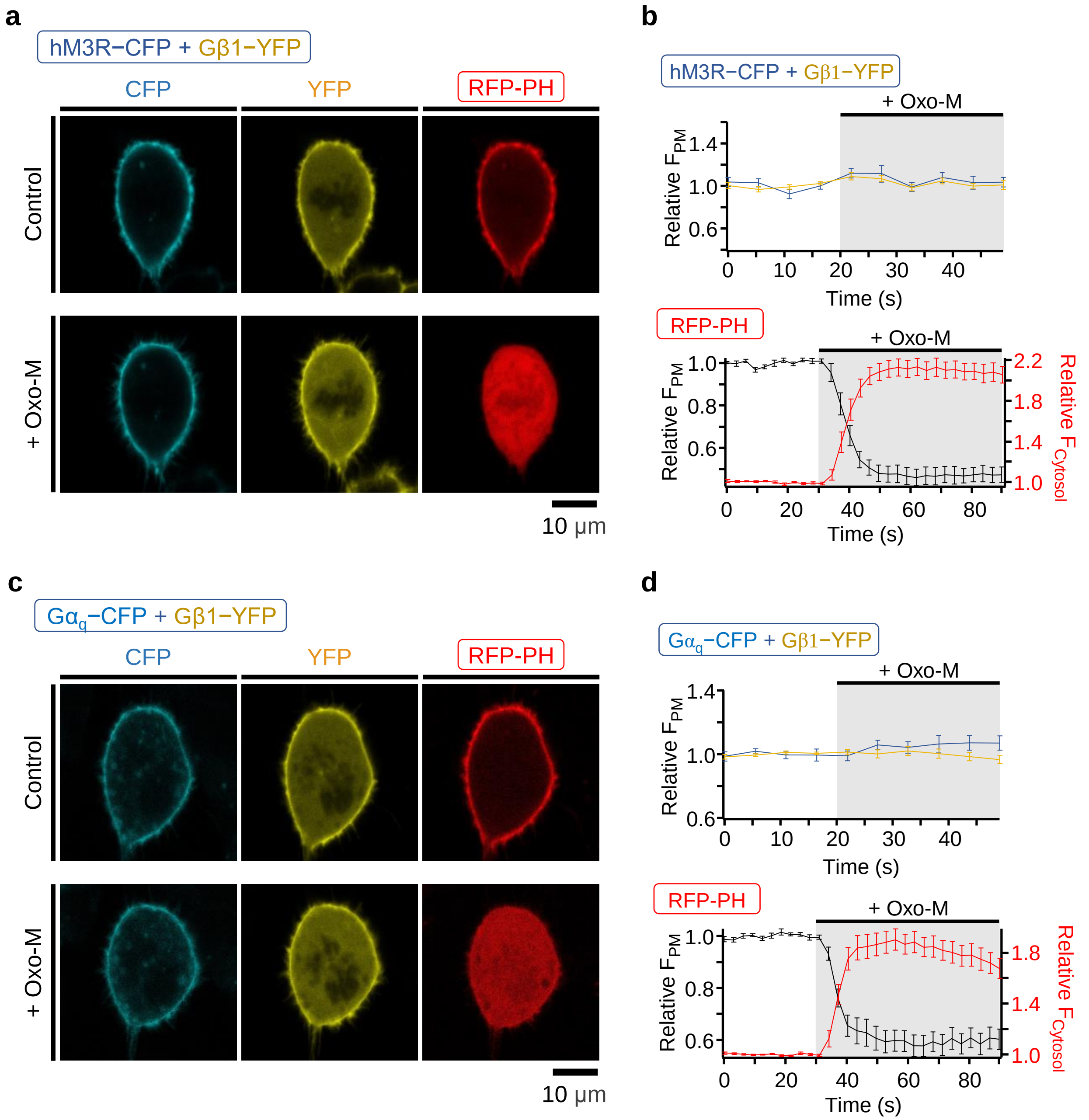

Supplementary Figure 7. Cells expressing FRET probes display active GPCR signaling. a, Representative confocal images of a cell expressing RFP-PH, hM3R-CFP, Gβ1-YFP and cognate G protein subunits were obtained before (Control) and during 10 μM Oxo-M application. Scale bar, 10 μm. **b**, Top, time courses of relative fluorescence intensity of hM3R-CFP (cyan trace) and Gβ1-YFP (yellow trace) at plasma membrane. n = 5 cells from two independent experiments. Sampling frequency: 0.2 Hz. Bottom, time courses of relative fluorescence intensity changes of RFP-PH at plasma membrane (black trace, left axis) and cytosol (red trace, right axis) in response to Oxo-M. n = 15 cells from two independent experiments. Sampling frequency: 0.33 Hz. **c**, Representative confocal images of a cell expressing RFP-PH, hM3R, $G\alpha_q$-CFP, Gβ1-YFP, Gγ2, and GRK2 were obtained before (Control) and during Oxo-M application. Scale bar, 10 μm. **d**, Top, time courses of relative fluorescence intensity of $G\alpha_q$-CFP (cyan trace) and Gβ1-YFP (yellow trace) at plasma membrane. n = 7 cells from two independent experiments. Sampling frequency: 0.2 Hz. Bottom, time courses of relative fluorescence intensity changes of RFP-PH at plasma membrane (black trace, left axis) and cytosol (red trace, right axis). n = 17 cells from two independent experiments. Sampling frequency: 0.33 Hz. Data are shown as mean ± SEM. Source data are provided as a Source Data file.

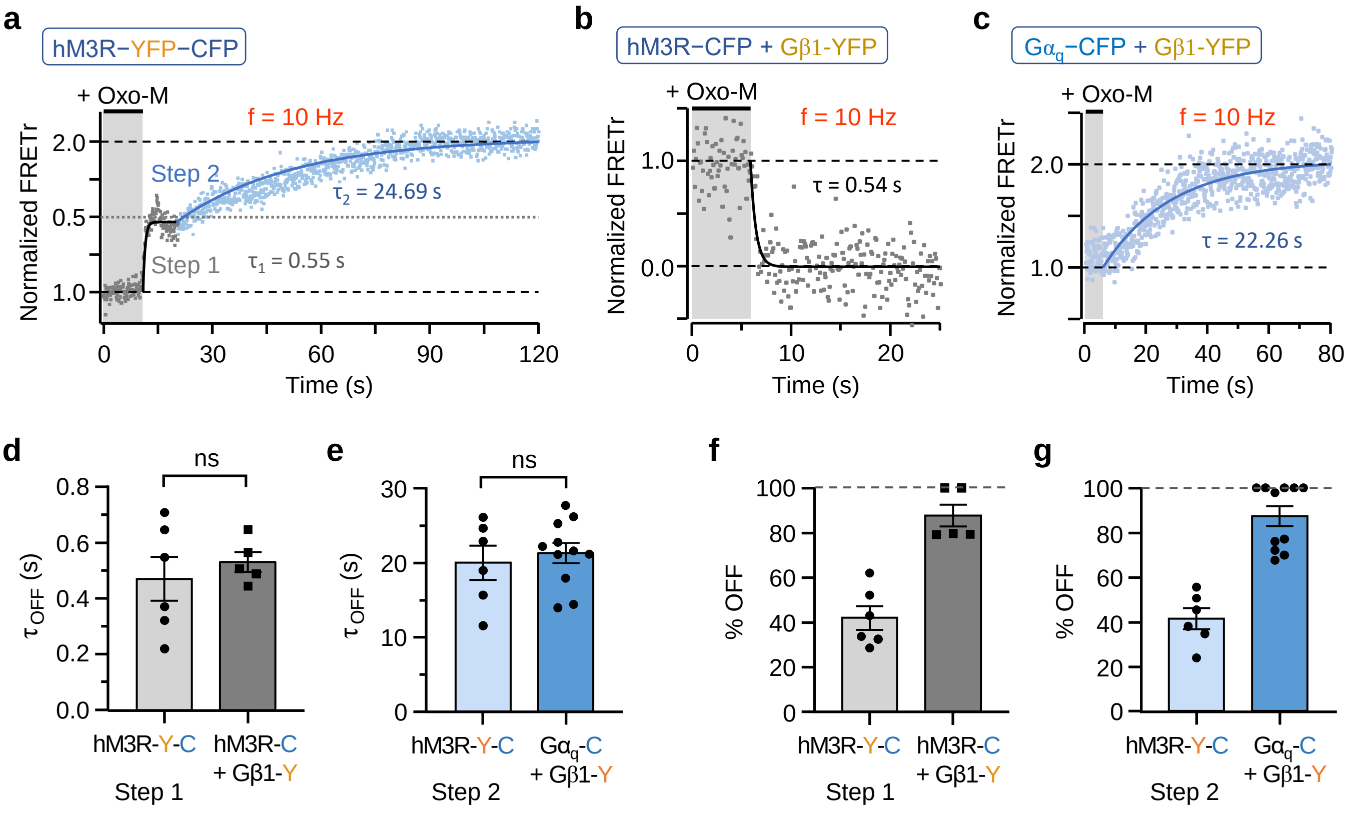

Supplementary Figure 8. hM3R-YFP-CFP FRET signal shows two-step deactivation. Representative time courses of normalized FRETr recovery from 10 μM Oxo-M-induced activation in cells expressing hM3R-YFP-CFP (**a**), hM3R-CFP, Gβ1-YFP and cognate G protein subunits (**b**), or hM3R, Gα$_q$-CFP, Gβ1-YFP, Gγ2 and GRK2 (**c**). Sampling frequency: 10 Hz. All black and blue lines are single-exponential fits with indicated τ values. **d**, Comparison of τ_{OFF} between hM3R-YFP-CFP (step 1) (n = 6 cells from four cultures) and hM3R-CFP plus Gβ1-YFP (n = 5 cells from two cultures). Statistical significance was determined using Student's t test (two-sided). **e**, Comparison of τ_{OFF} between hM3R-YFP-CFP (step 2) (n = 6 cells from four cultures) and Gα$_q$-CFP plus Gβ1-YFP (n = 11 cells from two cultures). Statistical significance was determined using Student's t test (two-sided). **f**, FRETr recovery (% OFF) between hM3R-YFP-CFP (step 1) (n = 6 cells from four cultures) and hM3R-CFP plus Gβ1-YFP (n = 5 cells from two cultures). **g**, FRETr recovery (% OFF) between hM3R-YFP-CFP (step 2) (n = 6 cells from four cultures) and Gα$_q$-CFP + Gβ1-YFP (n = 11 cells from two cultures). Data are shown as mean ± SEM. ns, not significant. Source data are provided as a Source Data file.

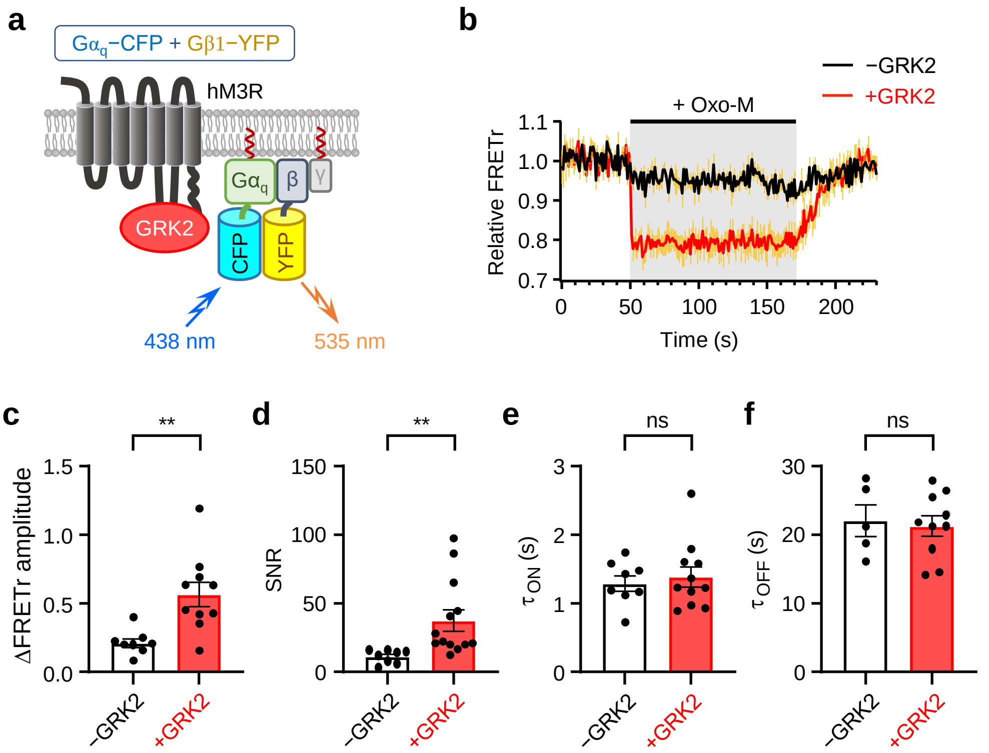

Supplementary Figure 9. GRK2 increases the amplitude of hM3R-mediated G-protein FRETr signal without change of kinetics. a, Cartoon of cells expressing hM3R, Gα_q-CFP, Gβ1-YFP, Gγ2 and GRK2. **b**, Time course of relative FRETr in response to Oxo-M in cells without (black trace, n=5) or with (red trace, n=10) GRK2. Yellow lines indicate SEM of the time points. **c**, Peak amplitude of FRETr changes of cells without and with GRK2. −GRK2, n = 8 cells from two cultures; +GRK2, n = 10 cells from two cultures. Statistical significance was determined using Welch's t test (two-sided, p = 0.0031). **d**, The signal to noise ratio (SNR) of ΔFRETr in the absence or presence of GRK2. −GRK2, n = 8 cells from two cultures; +GRK2, n = 10 cells from two cultures. Statistical significance was determined using Welch's t test (two-sided, p = 0.0068). **e**, Time constant (τ_{ON}) of Oxo-M-induced FRETr activation in the absence or presence of GRK2. −GRK2, n = 8 cells from two cultures; +GRK2, n = 10 cells from two cultures. Statistical significance was determined using Student's t test (two-sided). **f**, Time constant (τ_{OFF}) of FRETr recovery from Oxo-M-induced activation in the absence or presence of GRK2. −GRK2, n = 8 cells from two cultures; +GRK2, n = 10 cells from two cultures. Statistical significance was determined using Student's t test (two-sided). Data are mean ± SEM. **P<0.01; ns, not significant. Source data are provided as a Source Data file.

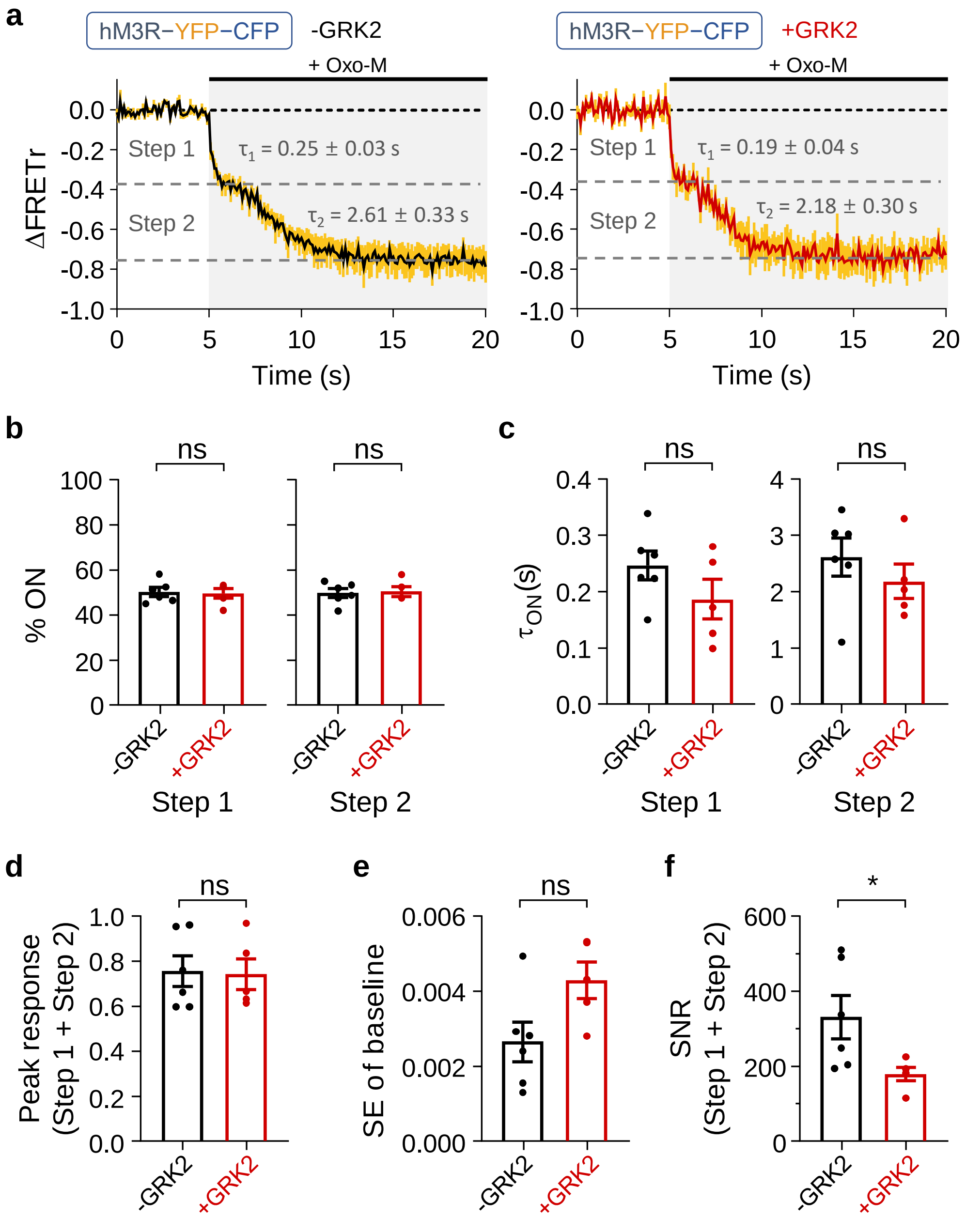

Supplementary Figure 10. GRK2 expression decreases the SNR of hM3R-YFP-CFP FRETr signal without change of kinetics and peak response. a, Time course of ΔFRETr in response to Oxo-M in cells without (black trace) or with (red trace) GRK2. Yellow lines indicate SEM of the time points. **b,** Percent distribution of each step (% of step 1_{ON} and step 2_{ON}) in the FRETr response. Statistical significances were determined using Student's t test (two-sided). **c**, Time constant (τ_{ON}) of Oxo-M-induced FRETr activation. Statistical significances were determined using Student's t test (two-sided). **d**, Peak amplitude of FRETr changes. Statistical significance was determined using Student's t test (two-sided). **e,** The standard error (SE) of baseline of ΔFRETr. Statistical significance was determined using Student's t test (two-sided). **f**, The signal to noise ratio (SNR) of ΔFRETr. −GRK2, n = 6 cells from two cultures; +GRK2, n = 5 cells from two cultures. Statistical significance was determined using Welch's t test (two-sided, p = 0.0450). Data are mean ± SEM. *P<0.05; ns, not significant. Source data are provided as a Source Data file.

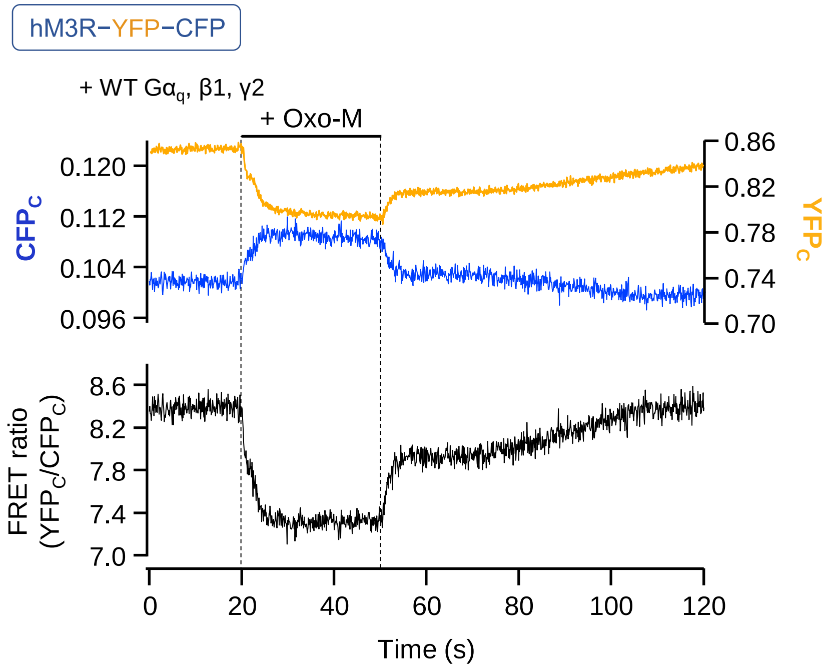

Supplementary Figure 11. Time-dependent changes of FRETr photometry signal by 10 μM Oxo-M in a single cell expressing hM3R-YFP-CFP plus wild-type G_q proteins (Gα_q, β1 and γ2). Top panel shows the fluorescence intensity changes of CFP_C (blue trace, left axis) and YFP_C (yellow trace, right axis), and bottom panel shows the FRET ratio (YFP_C/CFP_C) (black). Sampling frequency: 10 Hz. Source data are provided as a Source Data file.

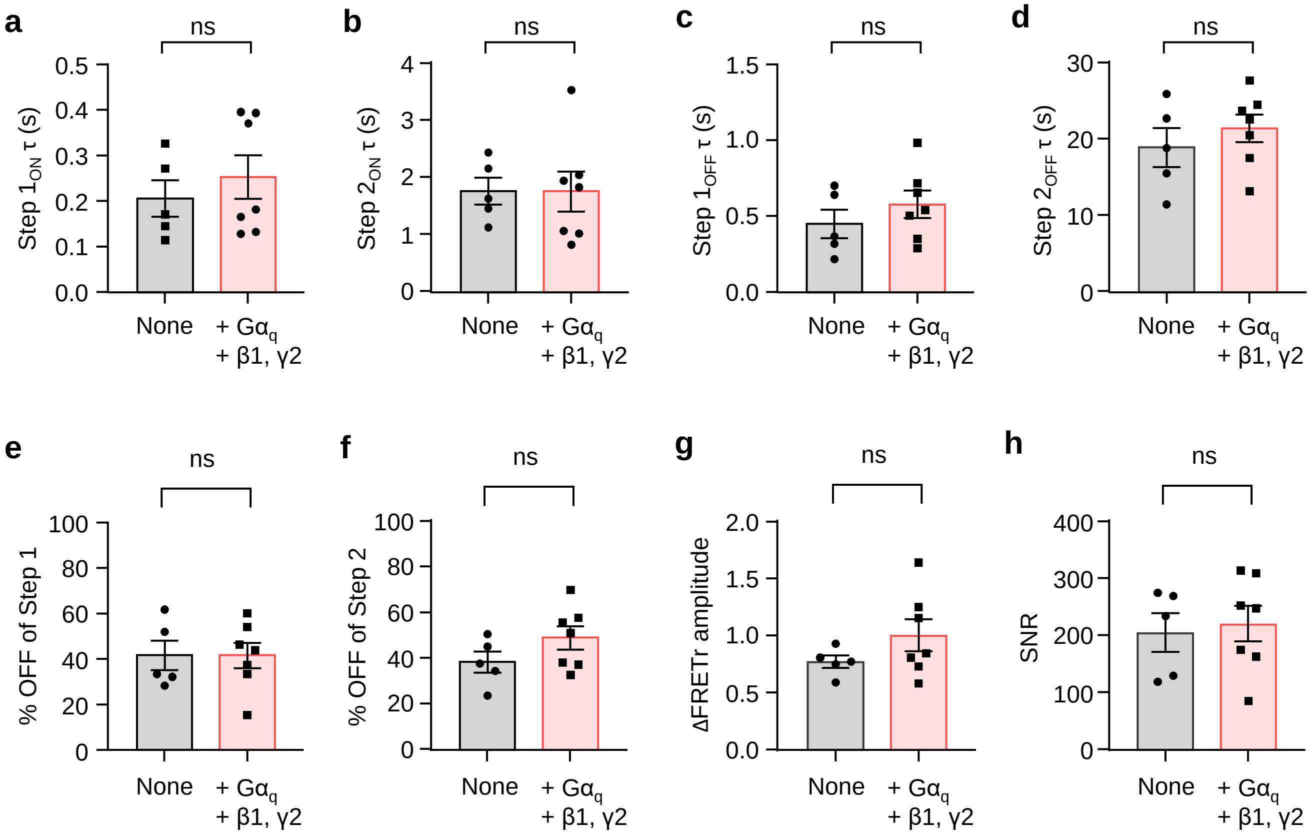

Supplementary Figure 12. Coexpression of wild type G_q protein subunits does not affect the FRETr responses of hM3R-YFP-CFP. Time constants (τ) of step 1_{ON} (**a**), step 2_{ON} (**b**), step 1_{OFF} (**c**) and step 2_{OFF} (**d**) were measured in cells transfected with hM3R-YFP-CFP alone (none) or hM3R-YFP-CFP plus wild-type G_q proteins ($G\alpha_q$, β1 and γ2). None, n = 5 cells from three cultures; + G proteins, n = 7 cells from three cultures. Statistical significances were determined using Student's t test (two-sided). Percent distribution (%) of step 1 (**e**) and step 2 (**f**) in the total recovery were measured in the two groups. None, n = 5 cells from three cultures; + G proteins, n = 7 cells from three cultures. Statistical significances were determined using Student's t test (two-sided). **g**, Peak response amplitude of ΔFRETr in cells with hM3R-YFP-CFP alone (none) or hM3R-YFP-CFP plus wild-type G_q proteins (+ $G\alpha_q$, β1 and γ2). None, n = 5 cells from three cultures; + G proteins, n = 7 cells from three cultures. Statistical significance was determined using Student's t test (two-sided). **h**, The signal to noise ratio of ΔFRETr in the two groups of cells. Sampling frequency: 10 Hz. None, n = 5 cells from three cultures; + G proteins, n = 7 cells from three cultures. Statistical significance was determined using Student's t test (two-sided). Data are shown as mean ± SEM. ns, not significant. Source data are provided as a Source Data file.

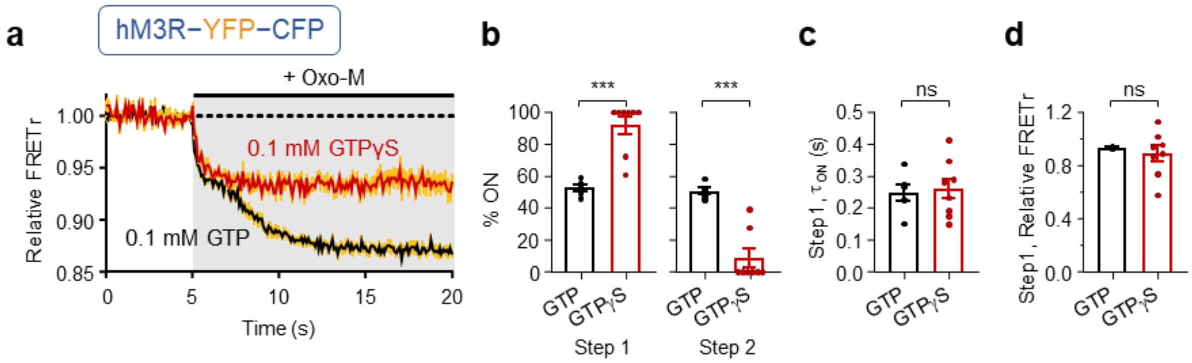

Supplementary Figure 13. Non-hydrolysable GTP analog, GTPγS has a similar effect to Gα_q(Q209L) in the FRET response of hM3R-YFP-CFP. a, Relative FRETr signal in hM3R-YFP-CFP expressing cells perfused with 0.1 mM GTP (black trace, n = 6 cells from three cultures) or GTPγS (red trace, n = 12 cells from two cultures). Sampling frequency: 10 Hz. Yellow vertical lines indicate SEM. n = 11 cells from two cultures. **b,** Percent distribution of each step (% of step 1_{ON} and step 2_{ON}) in the FRETr response under perfused conditions. GTP, n = 6 cells from three cultures; GTPγS, n = 12 cells from two cultures. Statistical significances were determined using Welch's t test (two-sided, p = 0.0001). **c,** Time constant (τ_{ON}) of step 1 under perfused conditions. GTP, n = 6 cells from three cultures; GTPγS, n = 12 cells from two cultures. Statistical significance was determined using Student's t test (two-sided). **d,** Relative FRETr of step 1 under perfused conditions. GTP, n = 6 cells from three cultures; GTPγS, n = 12 cells from two cultures. Statistical significance was determined using Welch's t test (two-sided). Data are shown as mean ± SEM. ***P<0.001; ns, not significant. Source data are provided as a Source Data file.

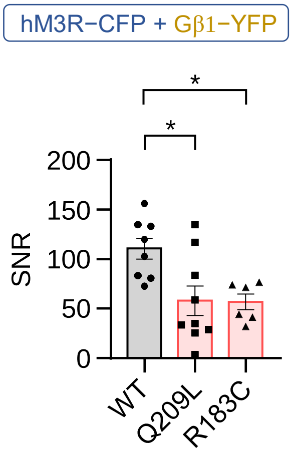

Supplementary Figure 14. Lower SNR of ΔFRETr between hM3R-CFP and Gβ1−YFP in cells co-expressing $G\alpha_q$(Q209L) or $G\alpha_q$(R183C) compared with the cells co-expressing wild-type $G\alpha_q$. The SNR of ΔFRETr in cells expressing hM3R-CFP, Gβ1−YFP, Gγ2 and wild-type or mutant (Q209L or R183C) $G\alpha_q$. WT, n = 8 cells from two cultures; Q209L, n = 9 cells from two cultures; R183C, n = 6 cells from two cultures. Statistical significance was determined using one-way ANOVA test with Tukey post hoc test. p = 0.0088; wild-type vs Q209L p = 0.0147, wild-type vs R183C p = 0.0253, Q209L vs R183C p = 0.9979. Data are shown as mean ± SEM. *P<0.05. Source data are provided as a Source Data file.

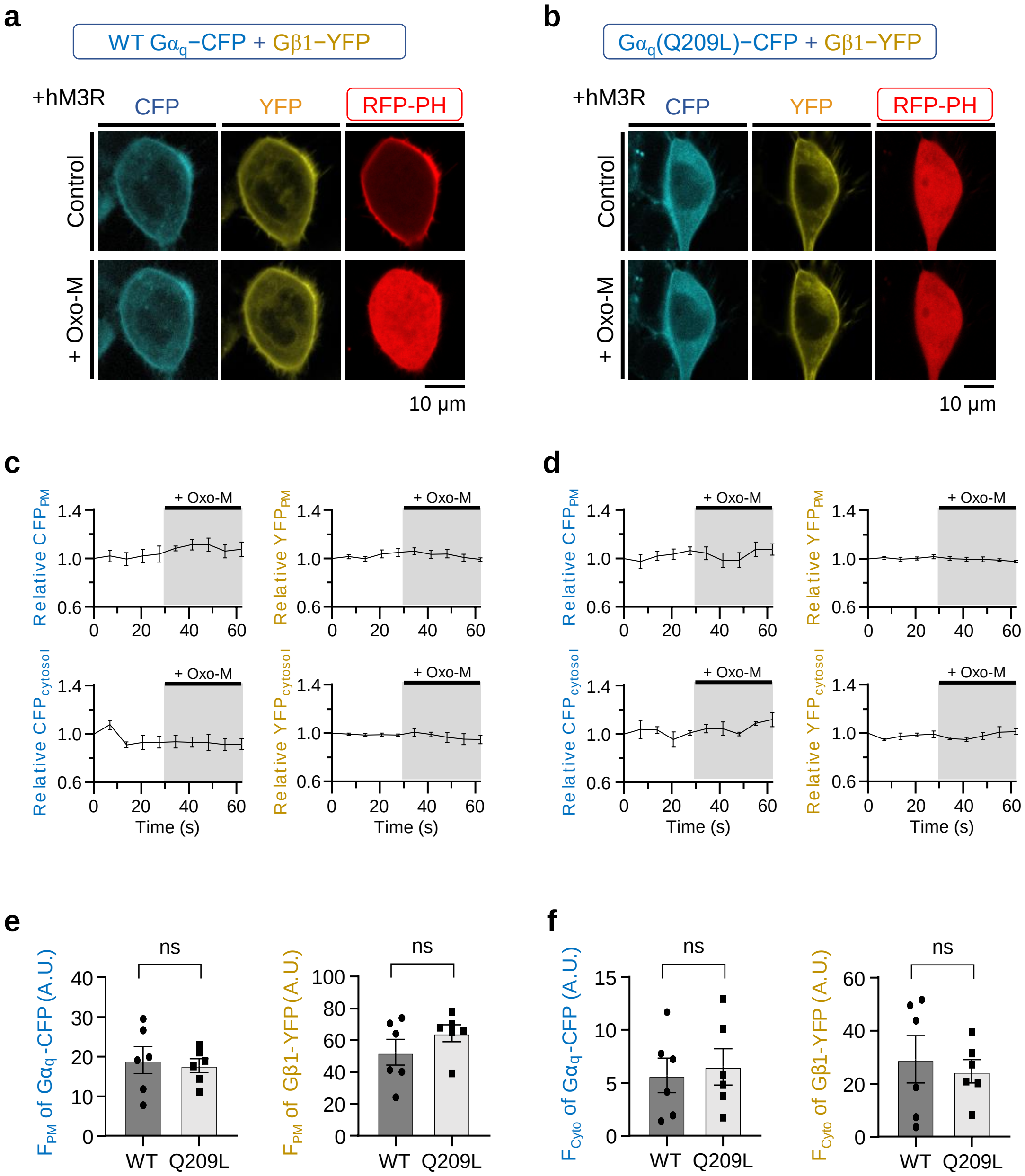

Supplementary Figure 15. Mutant Gα_q(Q209) constitutively activates downstream signals. a-b, Representative confocal images of a cell expressing RFP-PH, hM3R, each type of Gα_q-CFP (wild type or Q209L), Gβ1-YFP, and Gγ2 were obtained before Oxo-M (10 μM) application (control) and during Oxo-M application (+ Oxo-M). Scale bar, 10 μm. **c-d**, Time courses of relative fluorescence intensity of Gα_q-CFP (wild type or Q209L) and Gβ1-YFP at plasma membrane (top) and cytosol (bottom) were measured in 6 cells from two independent experiments. Sampling frequency: 0.2 Hz. **e**, Absolute fluorescence intensity of Gα_q-CFP and Gβ1-YFP at plasma membrane (WT, n = 6 cells from two cultures; Q209L, n = 6 cells from two cultures) according to the type of Gα_q-CFP. Statistical significances were determined using Student's t test (two-sided). **f**, Absolute fluorescence intensity of Gα_q-CFP and Gβ1-YFP at cytosol (WT, n = 6 cells from two cultures; Q209L, n = 6 cells from two cultures) according to the type of Gα_q-CFP. Statistical significances were determined using Student's t test (two-sided). Data are shown as mean ± SEM. ns, not significant. Source data are provided as a Source Data file.

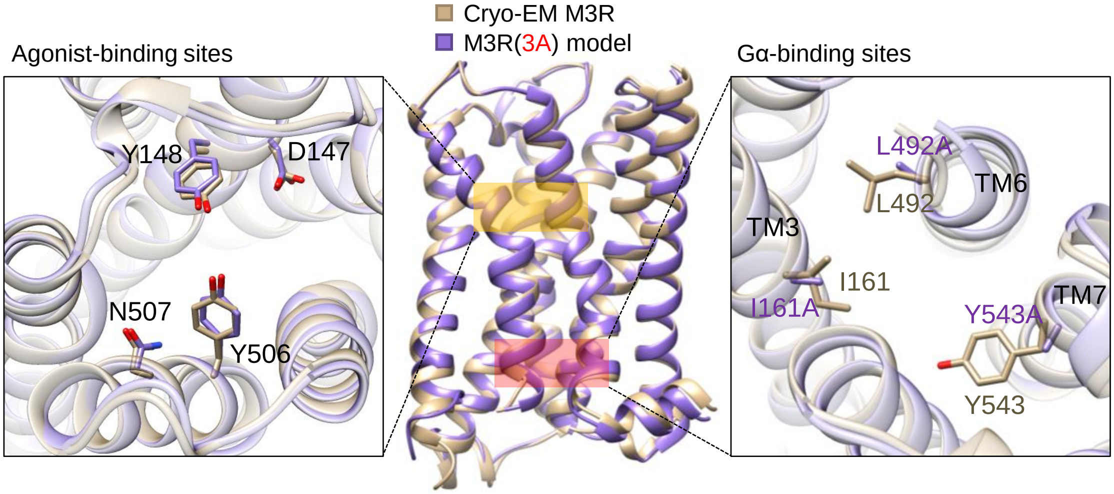

Supplementary Figure 16. Comparison of cryo-EM rat M3R (PDB ID : 4DAJ)[53] and SWISS-Model 3D predicted M3R(3A) on orthosteric agonist-binding sites (left) and Gα-binding sites (right, 3x46 (I161), 6x37 (L492), 7x53 (Y543)). The yellow and red boxes in the middle panel indicate agonist- and Gα-binding regions of M3R, respectively. Amino acid residues on binding sites are shown as a stick in tan (cryo-EM M3R) or purple (SWISS-Model simulation).

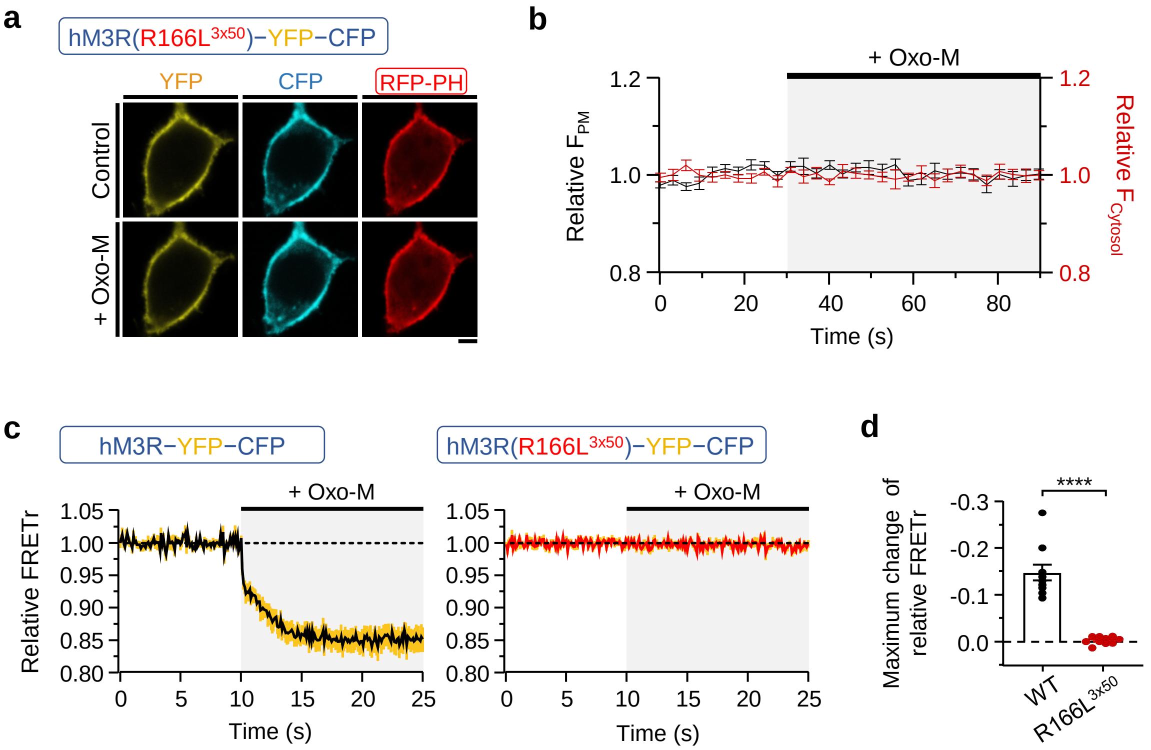

Supplementary Figure 17. R166L^{3x50} blocks G_q signaling of hM3R-YFP-CFP. a, Representative confocal images of a cell expressing red fluorescent protein-labelled pleckstrin homology domain of PLCδ1 (RFP-PH) and inactive hM3R(R166L^{3x50})-YFP-CFP were obtained before (control) and during 10 μM Oxo-M application. Scale bar, 5 μm. **b,** time courses of relative fluorescence intensity of RFP-PH at plasma membrane (black trace, left axis) and cytosol (red trace, right axis) were measured in 10 cells from two independent experiments. Sampling frequency: 0.33 Hz. **c,** Time course of relative FRETr in response to 15 s of 10 μM Oxo-M in cells expressing wild-type hM3R-YFP-CFP (left) or hM3R(R166L^{3x50})-YFP-CFP (right). Sampling frequency: 10 Hz. Yellow vertical lines indicate SEM. hM3R-YFP-CFP(WT), n = 10 cells from two cultures; hM3R(R166L^{3x50})-YFP-CFP, n = 10 cells from two cultures. **d,** Maximum change of relative FRETr in cells expressing each FRET sensor by 10 μM Oxo-M treatment. hM3R-YFP-CFP(WT), n = 10 cells from two cultures; hM3R(R166L^{3x50})-YFP-CFP, n = 10 cells from two cultures. Statistical significance was determined using Welch's t test (two-sided, p < 0.0001). Data are shown as mean ± SEM, with error bars indicating SEM. ****P<0.0001. Source data are provided as a Source Data file.

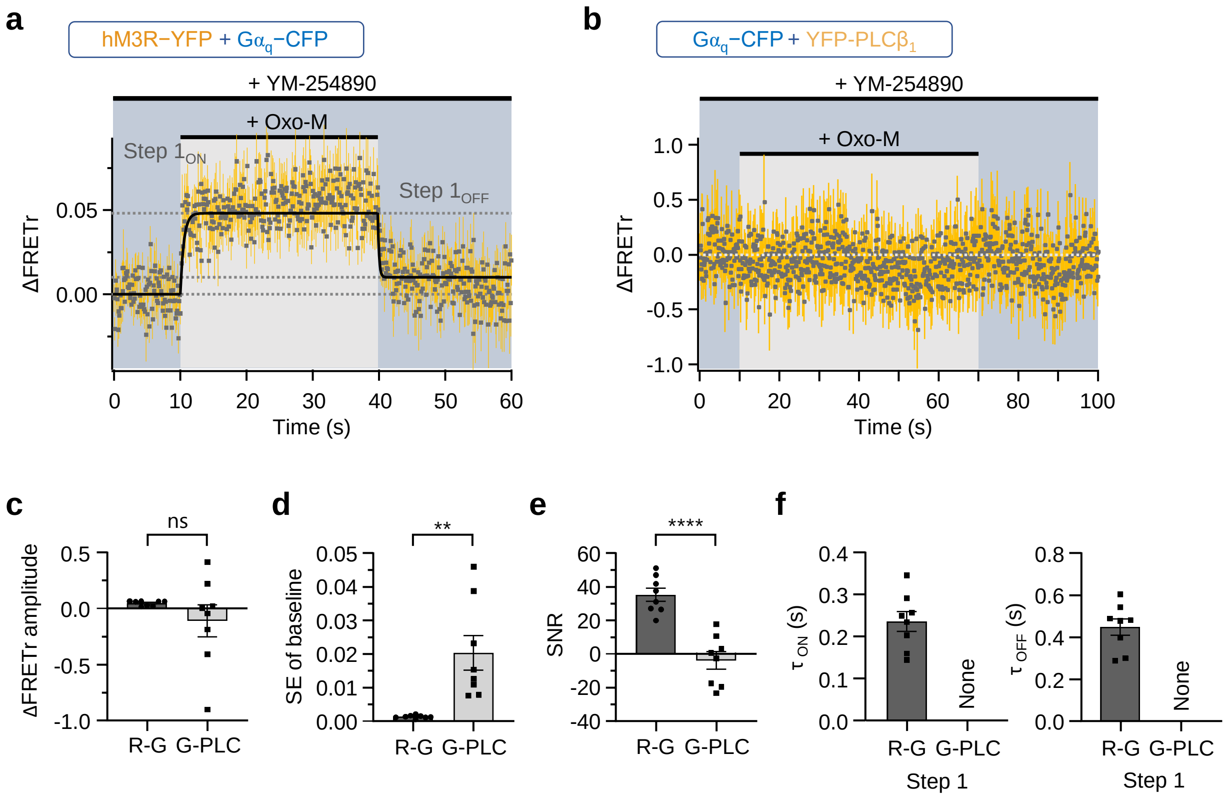

Supplementary Figure 18. YM-254890 inhibits the interaction between Gα_q and PLCβ_1 during receptor activation. Mean time course of ΔFRETr in response to 30-s or 60-s Oxo-M in cells expressing hM3R-YFP, Gα_q-CFP, and cognate G protein subunits (R-G) **(a)** or in cells expressing hM3R, Gα_q-CFP, YFP-PLCβ_1, and cognate G protein subunits (G-PLC) **(b)** in the presence of YM-254890 (YM). n = 8 cells from two cultures for R-G; n = 8 cells from two cultures for G-PLC. Sampling frequency: 10 Hz. Yellow lines are SEM. **c**, Amplitude of ΔFRETr in cells expressing each FRET sensor. n = 8 cells from two cultures for R-G; n = 8 cells from two cultures for G-PLC. Statistical significance was determined using Welch's t test (two-sided). **d**, Standard error of ΔFRETr baseline. n = 8 cells from two cultures for R-G; n = 8 cells from two cultures for G-PLC. Statistical significance was determined using Welch's t test (two-sided, p = 0.0081). **e,** Signal to noise ratio (SNR) of ΔFRETr. n = 8 cells from two cultures for R-G; n = 8 cells from two cultures for G-PLC. Statistical significance was determined using Student's t test (two-sided, $p < 0.0001$). **f**, Time constants (τ_{ON} and τ_{OFF}) of step 1_{ON} and step 1_{OFF}. n = 8 cells from two cultures for R-G. Data are shown as mean ± SEM, with error bars indicating SEM. **P<0.01; ****P<0.0001; ns, not significant. Source data are provided as a Source Data file.

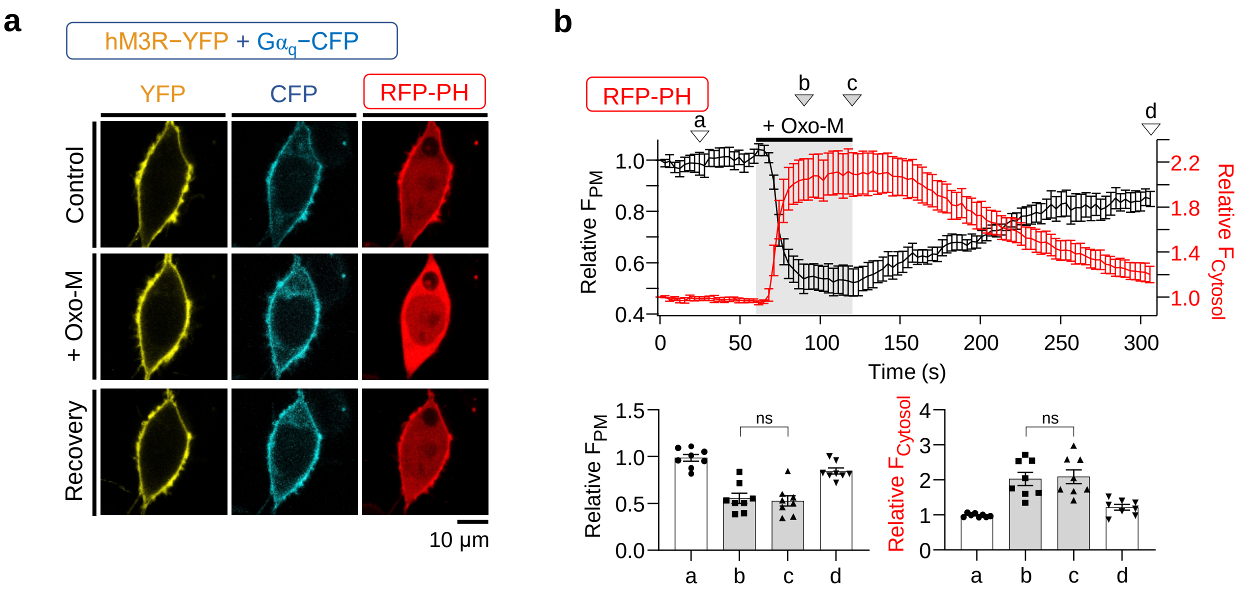

Supplementary Figure 19. Normal M3R signaling in cells expressing hM3R-YFP and Gα$_q$-CFP. a, Representative confocal images of a cell expressing RFP-PH, hM3R-YFP, Gα$_q$-CFP, Gβ1, and Gγ2 were obtained before (control), during (+ Oxo-M) and after (recovery) 10 µM Oxo-M application. Scale bar, 10 µm. **b**, Top, time courses of relative fluorescence intensity changes of RFP-PH at plasma membrane (black trace, left axis) and cytosol (red trace, right axis) in response to Oxo-M. Bottom, average of relative fluorescence intensity of RFP-PH at plasma membrane (left graph) and cytosol (right graph) at each marked time period (a: 19-25 s; b: 84-90 s; c: 114-120 s; d: 300-306 s) in top figure. n = 8 cells from two independent experiments. Sampling frequency: 0.33 Hz. Statistical significances were determined using Paired t test (two-sided). Data are shown as mean ± SEM. ns, not significant. Source data are provided as a Source Data file.

Supplementary Table 1. Primers for plasmid cloning

Name of Plasmid	Mutagenesis & Cloning experiments	Forward primer (5′ → 3′)	Reverse primer (5′ → 3′)
wild type hM3R from hM3D	C149Y mutation	GCTGGCTACGTAGTCAATGGCAAGCCAGAG	CTCTGGCTTGCCATTGACTACGTAGCCAGC
	G239A mutation	GACAGGCATATAAAAAGCAGCGATGGCTGTGCC	GGCACAGCCATCGCTGCTTTTTATATGCCTGTC
hM3R-Cerulean	(restriction enzyme sites for Cerulean tagging) *Bam*HI-hM3R-*Eco*RI	GATTACGCTGGATCCATGACCTTGCA	GATCCAACTAGAATTCCAAGGCCTGC
hM3R-EYFP	(restriction enzyme sites for EYFP tagging) *Eco*RI-EYFP-*Not*I	GAGCAGGCCTTGGAATTCATGGTGAGCAAGGGCGAGGA	ATGCATGCTCGAGCGGCCGCTTACTTGTACAGCTCGTCCA
hM3R-EYFP-Cerulean	(restriction enzyme sites for EYFP insertion) hM3R-*Sac*II-*Age*I-Cerulean	(*Sac*II) CCGCGGTCTGGGACAGAGGCAGAGAC	(*Sac*II) GGCTTGCAGGCCAGCAAGCTCTTTGG
		(*Age*I) GCCCAGACCCTCAGTGCGATCTTGCTTGCCTTCATC	(*Age*I) CGCTTTCTTCTCCTTACCGGTGACCAGGGACATCC
	(restriction enzyme sites for EYFP insertion) *Sac*II-EYFP-*Age*I	CCGCGGATGGTGAGCAAGGGCGAGGA	ACCGGTCTTGTACAGCTCGTCCATGCCGA
Gα_q(Q209L)	Q209L mutation	GATGTAGGGGGCCTAAGGTCAGAGAG	CTCTCTGACCTTAGGCCCCCTACATC
Gα_q(Q209L)-ECFP		ATGTAGGGGGCCTAAGGTCAGAGAG	CGACCATTCTGAAAATGACACTTTG
Gα_q(R183C)	R183C mutation	CAACAAGATGTGCTTAGAGTTTGTGTCCCCACCACAGGGATCATC	GATGATCCCTGTGGTGGGGACACAAACTCTAAGCACATCTTGTTG
Gα_q(R183C)-ECFP		GTGCTTAGAGTTTGTGTCCCCACTACA	GTCTTGCTCTGTCGGCATGTACTCA
hM3R-EYFP (3A), hM3R-Cerulean (3A)	I162A mutation	AATCTTCTGGTCGCTAGCTTTGACAGA	CATAACAGAGGCATTGCTGGCTAC
	L493A mutation	GCGGCCCAGACCGCTAGTGCGATCTTG	TTTCTTCTCCTTGACCAGGGACAT
	Y544A mutation	AACCCCGTGTGCGCTGCTCTGTGCAAC	CACGGTGCTGTTGATGTAGCACAG
hM3R(3A)-EYFP-Cerulean	I162A mutation	AATCTTCTGGTCGCTAGCTTTGACAGA	CATAACAGAGGCATTGCTGGCTAC
	L493A mutation	GCGGCCCAGACCGCTAGTGCGATCTTG	TTTCTTCTCCTTACCGGTCTTGTA
	Y544A mutation	AACCCCGTGTGCGCTGCTCTGTGCAAC	CACGGTGCTGTTGATGTAGCACAG
hM3R(R166L^{3x50})-EYFP-Cerulean	R166L mutation	ATCAGCTTTGACCTATACTTTTCCATC	GACCAGAAGATTCATAACAGAGGC
