# Supplementary material for: Two-step structural changes in M3 muscarinic receptor activation rely on the coupled Gq protein cycle
Source: Nat Commun. 2023 Mar 8;14:1276. doi: 10.1038/s41467-023-36911-4 (PMC9992711; doi:10.1038/s41467-023-36911-4)
Supplement: Supplementary file 2 — Reporting Summary [file 41467_2023_36911_MOESM2_ESM.pdf]

nature portfolio

Corresponding author(s): Byung-Chang Suh

Last updated by author(s): Jan 27, 2023

Reporting Summary

Nature Portfolio wishes to improve the reproducibility of the work that we publish. This form provides structure for consistency and transparency in reporting. For further information on Nature Portfolio policies, see our Editorial Policies and the Editorial Policy Checklist.

Statistics

For all statistical analyses, confirm that the following items are present in the figure legend, table legend, main text, or Methods section.

n/a	Confirmed	
☐	☒	The exact sample size (*n*) for each experimental group/condition, given as a discrete number and unit of measurement
☐	☒	A statement on whether measurements were taken from distinct samples or whether the same sample was measured repeatedly
☐	☒	The statistical test(s) used AND whether they are one- or two-sided<br>*Only common tests should be described solely by name; describe more complex techniques in the Methods section.*
☒	☐	A description of all covariates tested
☐	☒	A description of any assumptions or corrections, such as tests of normality and adjustment for multiple comparisons
☐	☒	A full description of the statistical parameters including central tendency (e.g. means) or other basic estimates (e.g. regression coefficient) AND variation (e.g. standard deviation) or associated estimates of uncertainty (e.g. confidence intervals)
☐	☒	For null hypothesis testing, the test statistic (e.g. *F*, *t*, *r*) with confidence intervals, effect sizes, degrees of freedom and *P* value noted<br>*Give P values as exact values whenever suitable.*
☒	☐	For Bayesian analysis, information on the choice of priors and Markov chain Monte Carlo settings
☒	☐	For hierarchical and complex designs, identification of the appropriate level for tests and full reporting of outcomes
☒	☐	Estimates of effect sizes (e.g. Cohen's *d*, Pearson's *r*), indicating how they were calculated

Our web collection on statistics for biologists contains articles on many of the points above.

Software and code

Policy information about availability of computer code

Data collection	ZEN 2010 (Carl Zeiss LSM 700 confocal imaging),<br>PatchMaster 2x90 (real time FRET signal acquisition)
Data analysis	I-TASSER server V5.2 (rat M3R (3A) structure prediction),<br>SWISS-Model web server released from July 2022 (rat M3R (3A) structure prediction)<br>UCSF Chimera 1.16 (visualization and modification of receptor structures),<br>Zeiss ZEN 2.3 (confocal image analysis),<br>Microsoft Excel 2016 (calculating the average and standard error of all data),<br>Igor Pro 6.0 (analysis of FRET data),<br>GraphPad Prism 8 (statistical analysis and graph processing).

For manuscripts utilizing custom algorithms or software that are central to the research but not yet described in published literature, software must be made available to editors and reviewers. We strongly encourage code deposition in a community repository (e.g. GitHub). See the Nature Portfolio guidelines for submitting code & software for further information.

Data

Policy information about availability of data

All manuscripts must include a data availability statement. This statement should provide the following information, where applicable:

- Accession codes, unique identifiers, or web links for publicly available datasets
- A description of any restrictions on data availability
- For clinical datasets or third party data, please ensure that the statement adheres to our policy

Source data are provided with this paper.
All data supporting the finding of this study are provided as a Source Data file.
The cryo-EM structure of rat M3R (PDB ID: 4DAJ [http://doi.org/10.2210/pdb4daj/pdb]).
The X-ray crystallized structure of rat M3R (PDB ID: 4U14 [http://doi.org/10.2210/pdb4U14/pdb]).

Human research participants

Policy information about studies involving human research participants and Sex and Gender in Research.

Reporting on sex and gender	No human research was performed.
Population characteristics	No human research was performed.
Recruitment	No human research was performed.
Ethics oversight	No human research was performed.

Note that full information on the approval of the study protocol must also be provided in the manuscript.

Field-specific reporting

Please select the one below that is the best fit for your research. If you are not sure, read the appropriate sections before making your selection.

☒ Life sciences ☐ Behavioural & social sciences ☐ Ecological, evolutionary & environmental sciences

For a reference copy of the document with all sections, see nature.com/documents/nr-reporting-summary-flat.pdf

Life sciences study design

All studies must disclose on these points even when the disclosure is negative.

Sample size	No sample size calculations were performed.<br>For imaging experiments, the number of single cell sample was chosen between 6 and 17 based on previous experiences and standards in the field (please refer following citations: https://doi.org/10.1073/pnas.1809762115, https://doi.org/10.1073/pnas.2014520117), after checking the localization patterns with the expressed protein throughout each cell.<br>For FRET experiments, the number of sample was chosen between 5 and 15 based on previous experiences and standards in the field (please refer to the References 74-76 of the manuscript). All group n>5 were considered for statistical comparison.
Data exclusions	No data points were excluded from the study.
Replication	The findings in this study were successfully reproducible. Every experiment was independently repeated with the similar results at least two to three time on different days.
Randomization	In all experiments, we randomly selected samples from cells expressing an adequate amount of fluorescent proteins.
Blinding	Investigators were not blinded during data collection and analysis. All experiments were performed using a machine and data are quantitative.

Reporting for specific materials, systems and methods

We require information from authors about some types of materials, experimental systems and methods used in many studies. Here, indicate whether each material, system or method listed is relevant to your study. If you are not sure if a list item applies to your research, read the appropriate section before selecting a response.

Materials & experimental systems

n/a	Involved in the study
☒	☐ Antibodies
☐	☒ Eukaryotic cell lines
☒	☐ Palaeontology and archaeology
☒	☐ Animals and other organisms
☒	☐ Clinical data
☒	☐ Dual use research of concern

Methods

n/a	Involved in the study
☒	☐ ChIP-seq
☒	☐ Flow cytometry
☒	☐ MRI-based neuroimaging

Eukaryotic cell lines

Policy information about cell lines and Sex and Gender in Research

Cell line source(s)	HEK293T cell line (RRID: CVCL_2737) was obtained from Bertil Hille (University of Washington School of Medicine, Seattle, Washington). The original source for this cell line is European Collection of Authenticated Cell Cultures (ECACC) (Cat# 96121229).
Authentication	The identity of HEK293T cell line was authenticated by STR profiling.
Mycoplasma contamination	HEK293T cell line was mycoplasma negative in the routine PCR-based mycoplasma detection tests.
Commonly misidentified lines (See ICLAC register)	No commonly misidentified cell lines were used.
